# Effectiveness of interventions to directly support food and drink intake in people with dementia: systematic review and meta-analysis

**DOI:** 10.1186/s12877-016-0196-3

**Published:** 2016-01-22

**Authors:** Asmaa Abdelhamid, Diane Bunn, Maddie Copley, Vicky Cowap, Angela Dickinson, Lucy Gray, Amanda Howe, Anne Killett, Jin Lee, Francesca Li, Fiona Poland, John Potter, Kate Richardson, David Smithard, Chris Fox, Lee Hooper

**Affiliations:** 1 Norwich Medical School, University of East Anglia, Norwich Research Park, Norfolk, NR4 7TJ UK; 2 Age UK Norfolk, 300 St Faith’s Road, Old Catton, Norwich, NR6 7BJ UK; 3 NorseCare, Lancaster House 16 Central Avenue St Andrew’s Business Park, Norwich, NR7 0HR UK; 4 School of Health and Social Work, University of Hertfordshire, Hatfield, Hertfordshire AL10 9AB UK; 5 School of Health Sciences, University of East Anglia, Norwich Research Park, Norfolk, NR4 7TJ UK; 6 Norfolk and Norwich University Hospital, Colney Lane, Norwich, NR4 7UY UK; 7 King’s College Hospital NHS Foundation Trust, Denmark Hill, London, SE5 9RS UK; 8 Norfolk and Suffolk NHS Foundation Trust, Hellesdon Hospital, Drayton High Road, Norwich, NR6 5BE UK; 9 Present address: Royal College of Paediatrics and Child Health, 5-11 Theobalds Road, London, WC1X 8SH UK

**Keywords:** Dementia, Aged, Eating, Drinking, Meta-analysis, Diet, Malnutrition, Dehydration

## Abstract

**Background:**

Eating and drinking difficulties are recognised sources of ill health in people with dementia. In the EDWINA (Eating and Drinking Well IN dementiA) systematic review we aimed to assess effectiveness of interventions to directly improve, maintain or facilitate oral food and drink intake, nutrition and hydration status, in people with cognitive impairment or dementia (across all settings, levels of care and support, types and degrees of dementia). Interventions included oral nutrition supplementation, food modification, dysphagia management, eating assistance and supporting the social element of eating and drinking.

**Methods:**

We comprehensively searched 13 databases for relevant intervention studies. The review was conducted with service user input in accordance with Cochrane Collaboration’s guidelines. We duplicated assessment of inclusion, data extraction, and validity assessment, tabulating data, carrying out random effects meta-analysis and narrative synthesis.

**Results:**

Forty-three controlled interventions were included, disappointingly none were judged at low risk of bias. Oral nutritional supplementation studies suggested small positive short term but unclear long term effects on nutritional status. Food modification or dysphagia management studies were smaller and of low quality, providing little evidence of an improved nutritional status. Eating assistance studies provided inconsistent evidence, but studies with a strong social element around eating/drinking, although small and of low quality provided consistent suggestion of improvements in aspects of quality of life. There were few data to address stakeholders’ questions.

**Conclusions:**

We found no definitive evidence on effectiveness, or lack of effectiveness, of specific interventions but studies were small and short term. People with cognitive impairment and their carers have to tackle eating problems despite this lack of evidence, so promising interventions are listed. The need remains for high quality trials tailored for people with cognitive impairment assessing robust outcomes.

**Systematic review registration:**

The systematic review protocol was registered (CRD42014007611) and is published, with the full MEDLINE search strategy, on Prospero [1].

**Electronic supplementary material:**

The online version of this article (doi:10.1186/s12877-016-0196-3) contains supplementary material, which is available to authorized users.

## Background

Over 47 million people are living with dementia worldwide [2], and age-standardized prevalence for people aged ≥60 years is 5-7 % [3, 4]. The incidence of dementia progressively increases with age, so that as populations become older prevalence rises, despite younger cohorts having a lower risk of dementia [5, 6]. As their disease progresses, the needs of people with dementia become increasingly complex: 76 % of institutionalised people with dementia needed to be fed, refused food or choked on liquid or solid foods [7]. Eating and drinking difficulties are a major source of ill health and stress for people living with dementia and their carers, and addressing these difficulties was identified as a top-ten research priority by people with dementia and their formal and informal carers [8], as well as an international survey of experts [9]. People with dementia are more likely to drink insufficient fluid, be malnourished, and malnutrition risk increases as dementia progresses [10–13]. While hospital admissions for dehydration and anorexia or malnutrition account for 1 % and 3 % of total hospital admissions, persons with dementia account for ten times more admissions when compared to age-matched controls [14]. Reasons may include poor appetite, decreased sense of thirst, increased fluid losses related to reduced urinary concentrating capacity, forgetting to eat and drink, days less structured around food and drink, reduced social contact, physical difficulties in shopping or food preparation, changes in food preferences, lack of formal or informal carer training and problems with chewing and swallowing [15–17]. The nature of difficulties vary with the stage of the illness.

This systematic review addresses these research priorities around eating and drinking in dementia by systematically reviewing existing research on direct and indirect interventions aiming to improve, maintain or facilitate the food or drink intake in adults with dementia at any age, at any stage (including mild cognitive impairment, MCI), and in any setting. Direct interventions aimed to modify food and/or drink, provide food or drink-based supplements, provide social support, assist with eating or drinking or manage swallowing problems, alone or as part of multi-component interventions. Indirect interventions included dining environment or food service modifications, educational, behavioral, exercise-type and multicomponent interventions. This paper reports on direct interventions, whilst the companion publication reports on indirect interventions [18]. No previous systematic review has addressed the full scope of interventions in people with this range of cognitive impairment. Key objectives included summarising evidence of effectiveness of interventions rigorously to minimise bias, addressing questions raised by our lay stakeholders (we involved stakeholders in designing the review to ensure we addressed questions relevant for people with dementia and their carers) and highlighting research priorities [8, 9, 19].

## Methods

We developed the systematic review protocol collaboratively, and the review team included lay stakeholders, subject experts and methodological experts. Lay stakeholders included members from AgeUK Norfolk and NorseCare (residential homes group). We worked with two patient and public involvement groups (the Public & Patient Involvement in Research, PPIRes, from Norfolk and Suffolk and the Public Involvement in Research Group, PIRG, from the University of Hertfordshire) to develop additional specific questions for the review. The protocol is published, with the full MEDLINE search strategy, on Prospero [1]. The review was conducted in accordance with Cochrane Collaboration’s guidelines [20], and reported in accordance with PRISMA guidance [21]. Study methods and specific questions posed by lay stakeholders are reported in full (Additional file 1), and summarised below.

### Criteria for inclusion

We included randomised (RCTs) and non-randomised (CCTs) intervention studies that fulfilled the following criteria:

- Participants: ≥3 adults diagnosed with any type/stage of dementia or mild cognitive impairment (MCI) or where the mean Mini Mental State Examination (MMSE) score plus one standard deviation was ≤26, in any setting.
- Duration: ≥5 consecutive days.
- Interventions: aimed to modify food and/or drink, provide food- or drink-based supplements, assist with eating or drinking or manage swallowing problems (pharmacological and pill-based supplements were excluded).
- Outcomes: nutrition or hydration status [22]; quantity, quality or adequacy of food or fluid intake, ability to eat independently, swallow without aspirating, enjoyment of food or drink or meaningful activity (activity around food or drink that is personally fulfilling, that people enjoy, look forward to or find important). Note - studies were only included if they collected at least one of these outcomes, but where studies were included we also extracted, and report, data provided on the following outcomes: quality of life, functional or cognitive status, views or attitudes, cost effectiveness, resource use, mortality and health outcomes.

### Search strategy

We developed a complex MEDLINE search strategy and adapted it for 12 further databases (EMBASE, CINAHL, PsychInfo, five Cochrane Databases, meta-register of controlled trials, ALOIS (Cochrane Dementia and Cognitive Improvement Group comprehensive register of dementia trials), Dissertation and Thesis abstracts, and International Alzheimer's Disease Research Portfolio (IADRP). Bibliographies of included studies and lists of included/excluded studies from relevant reviews were checked [23–28]. There were no language or date limitations.

### Study selection and data collection

Inclusion was assessed by two reviewers independently. Titles and abstracts were screened and full-text papers obtained if either reviewer considered it potentially eligible; full text papers were grouped into studies, and the review assessed interventions (some studies tested more than one intervention, while some interventions were described in several published papers) and assessed for inclusion. Corresponding authors were contacted where papers were published in languages other than English or there were insufficient data to assess suitability for inclusion or outcomes.

Data (publication details, participants, intervention, comparison, outcomes as above plus quality of life, functional or cognitive status, views or attitudes, cost effectiveness, resource use, mortality, health outcomes) and quality characteristics were extracted independently by two reviewers, discrepancies were resolved through discussion. Methodological quality was assessed using Cochrane risk of bias tool [20, 29]. In addition to generic criteria, we assessed funding bias, validity of dementia diagnosis, outcome measures and baseline comparability between groups. We considered a study at low risk of bias where it was at low risk of both selection biases (was randomised and had appropriate allocation concealment) and detection bias (blinding of outcome assessment).

### Data synthesis

Studies were grouped by type of intervention then study design (RCT/ CCT) for tables and narrative synthesis. CCTs included non-randomised studies with a concurrent comparator group and before-after (pre-post) studies, collectively referred to as CCTs. Random-effects meta-analysis of RCTs using Review Manager (RevMan 5.3) software was conducted where studies were suitably comparable (CCTs were summarised narratively). Heterogeneity was quantified using I^2^ [20]. As interventions are most useful if they have a lasting effect, subgrouping was by time from inception of the intervention to assessment of effects (≤12 weeks, >12-26 weeks, >26 weeks).

Secondary analyses used subgrouping to address additional questions formulated by lay stakeholders (Table 1), and an additional question raised while conducting the review, on whether interventions that focussed on improving social contact of people with dementia, in the context of food and drink, were successful in supporting review outcomes.

**Table 1:** Specific review questions formulated by members of the lay stakeholders, and the evidence found to address these questions

Area	Questions from lay stakeholders	Review findings
*Type of dementia*	For people with different types of dementia (Alzheimer’s, vascular, dementia with Lewy bodies, other types or mixed types), what interventions can help to maintain or improve food intake or nutritional status and fluid intake or hydration status?	Less than half of studies indicated type of dementia of participants, but most that did enrolled people with AD. Results of 8 ONS studies including AD patients were not consistent - some studies reported improvement in nutritional status or intake, others no effect. Studies of other interventions were too few to compare or inform conclusions.
*Stage of dementia*	What interventions can help to maintain or improve food intake or nutritional status and fluid intake or hydration status in people with mild cognitive impairment, mild/moderate/severe dementia?	Less than half of the studies had any data on stage of dementia of participants. Potential interventions are shown in Table 4, but in studies of people with mild, moderate and/or severe dementia oral nutritional supplements (ONS) improved one or more markers of nutritional status (though usually not all markers, and only over short periods of time). Three studies of fruit juice supplements in people with mild cognitive impairment showed little effect. Shared mealtimes with staff and a commercial lyophilised food also appeared to improve some markers of nutritional status in people with severe dementia, while social interventions (supporting social interactions around food and drink) appeared to improve measures of participation, interactions, happiness, autonomy and involvement in people with mild, moderate and severe dementia.
*Setting*	1. For people with dementia living in residential care or residing in a medical setting, what interventions can help to maintain or improve food intake or nutritional status and fluid intake or hydration status? 2. For people with dementia living in their own homes with or without a carer (full-time or occasional; close relative or paid carer), what interventions can help to maintain or improve food intake or nutritional status and fluid intake or hydration status?	Most of the studies were conducted in various residential or nursing settings, and very few in participants own homes. Generally, effectiveness of interventions related to the effectiveness of interventions in residential settings.
*Emotional and social issues*	For people with dementia, does emotional closeness of the carer (e.g. close relative vs paid carer) affect the outcomes?	Emotional closeness to the carer was not ever reported in studies, and carers generally appeared to be professional rather than family carers.
*Meaningful Activity*	1. For people with dementia, what interventions aimed at improving or maintaining food and/or fluid intake, nutrition or hydration status, support meaningful activity (activity around food or drink that is personally fulfilling, that people enjoy, look forward to or find important)? 2. For people with dementia, are there any interventions that decrease food or fluid intake, diminish enjoyment or quality of life, or diminish meaningful activity or social inclusion?	Few studies measured quality of life or happiness using a validated scale. However, some studies especially those with a strong social element (see main review) reported improved autonomy, involvement and interest of participants. Few interventions reported diminished intake or any poorer outcomes, except for a study that gave supplemental yogurt at breakfast, which resulted in reduced weight (possibly as the result of replacing rather than supplementing usual food)
*Individualised interventions*	Do individualised interventions appear more effective than those that are not individualised, in helping people with dementia to maintain or improve food and/or drink intake, nutrition or hydration status (or related outcomes)?	Studies of ONS did not offer individualised interventions (based on needs and preferences of participants) beyond a choice of flavours, but the one study of individualised snacks did not suggest they were helpful. Multicomponent individualised interventions were more positive, suggesting useful effects on some nutritional outcomes. Individualised dysphagia diet and a multicomponent food modification diet appeared to improve weight, and individualised eating assistance was not clearly helpful.
*Interventions around swallowing and oral hygiene*	1. Do interventions to assess swallowing (and where necessary treat swallowing problems) have any effect on food or drink intake, nutrition or hydration status (or related outcomes)? 2. Do interventions to improve oral hygiene have any effect on food or drink intake, nutrition or hydration status (or related outcomes)?	Studies assessing interventions for swallowing problems were generally inconclusive except that individual and multicomponent interventions including food modification appeared helpful in supporting nutritional status in several studies. No interventions aimed to improve oral hygiene.
*Interventions in acute illness*	Are there any interventions that are particularly effective in helping people with dementia to maintain or improve food and/or drink intake, nutrition or hydration status (or related outcomes) during periods of acute illness?	Only one study included people with acute illness. It provided ONS during acute illness and reported no change in nutritional status [52].

What are the most effective ways to encourage people with dementia to eat, drink and maintain nutritional intake? Information provided here is supplemental to the main findings of this review, and overall evidence is weak or lacking – the review does not definitively show that any intervention is either useful or not useful

## Results

**Fig. 1:**
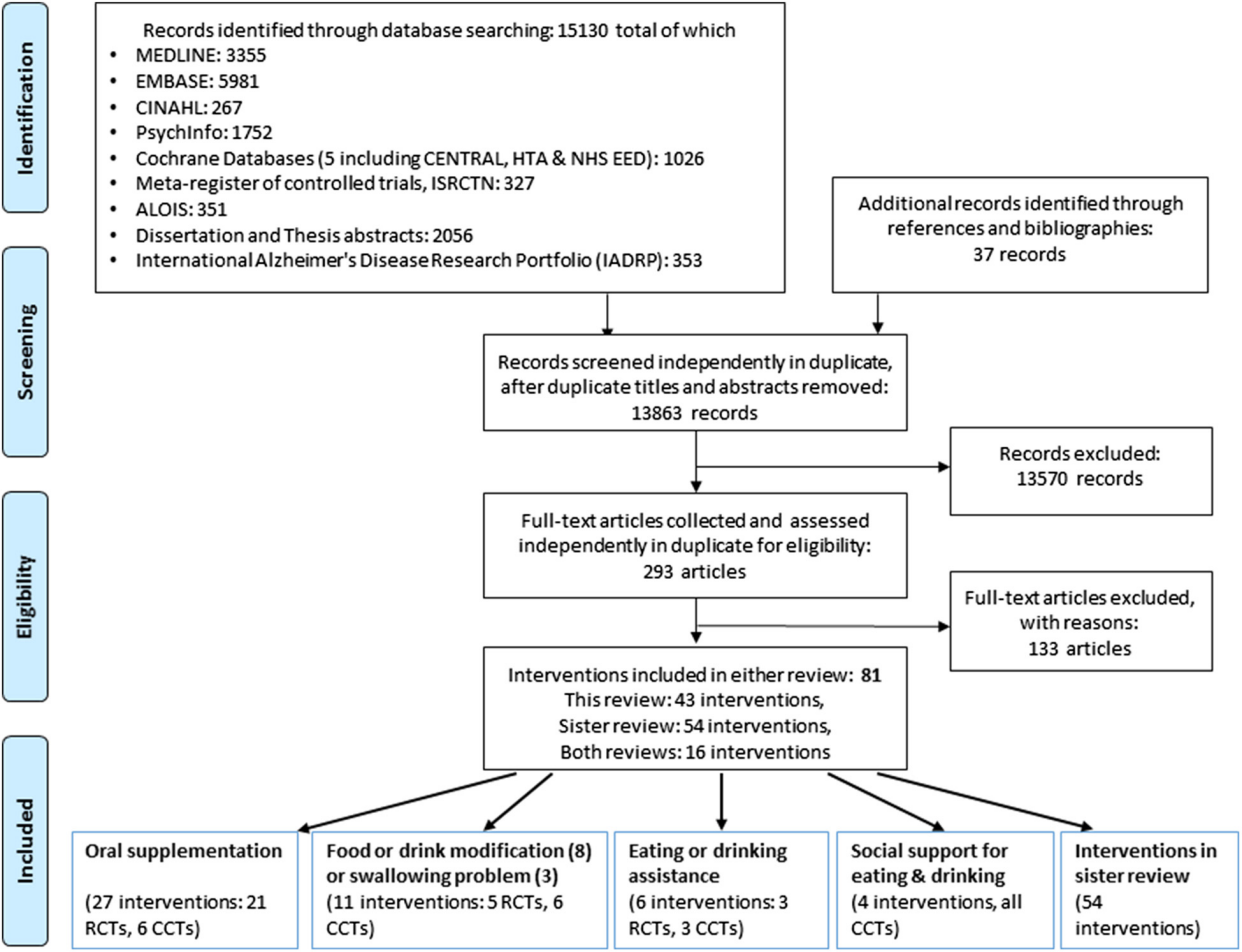
EDWINA systematic review PRISMA flow diagram for studies of direct interventions*. *The number of interventions by category adds up to more than 43 (the total number of interventions in this paper) as several interventions were multicomponent, and so represented in several categories

Searches identified 15468 citations (references and bibliographies added 37). After de-duplication we assessed 13863 titles and abstracts, and collected 293 full text papers for further assessment. Forty three direct interventions were included (Fig. 1).

Included interventions were from Europe (22 interventions), North America (17), Europe and the USA (multi-national study, one intervention, Brazil, Taiwan and New Zealand (one each). Most interventions (36) were conducted in institution or hospital settings, with four in day centres or the community, and unclear setting in one. Twenty eight interventions stated that participants were diagnosed with dementia, three with MCI, and in 12 cognitive impairment was assumed from cognitive scores or setting. Dementia staging was possible in 19 interventions, of which four were severe, two moderate, three mild dementia, three MCI, two moderate to severe, two mild to moderate and three mixed. Type of dementia was not always specified, but 13 interventions included participants with Alzheimer type dementia (AD) and eight included people with various dementia types.

Direct interventions were categorised into: oral supplements, food/drink modification, swallowing problems management, eating assistance and social support. Findings are presented under these headings below. Study characteristics and results are summarised in Tables 2 and 3, for full details of included studies see Additional file 2.

**Table 2:** Characteristics and results of included oral nutrition supplementation (ONS) interventions

Study	Design	Setting, supplement type	Number completed	Dementia stage	Dementia type	Effect on nutrition or hydration status	Effect on intake of nutrients or fluid	Quality and Other outcomes	Duration
ONS (including energy, protein and often other nutrients) plus usual food vs. usual food (with or without placebo ONS)									
Abalan 1992 France [30]	RCT	Geriatric inpatients. Proprietary ONS (‘Tonexis’) vs usual food	I = 15 C = 14	NR	AD	N/A	→ E intake	↑ Cognitive function	15 weeks
Beck 2002 Denmark [32]	RCT	Nursing home (risk of malnourishment). Home-made ONS vs usual food	I = 8 C = 8	NR	NR	→ Weight	→ E intake	N/A	2 months
Carlsson 2009 Sweden [34]	CCT (BA)	Group-living facilities for people with dementia. Drinkable yogurt	13	NR	Mixed	↓ Weight	→ E intake → Fluid intake	→ Functional status	6 months
Carver 1995 UK [36]	RCT	Psychiatric hospital/elderly ward (under-weight). Proprietary ONS (‘Fortisip’) vs placebo	I = 20 C = 20	NR	NR	↑ Weight ↑* BMI →TSF ↑ MAMC	N/A	N/A	12 weeks
de Sousa 2012 Portugal [37]	RCT	Psychiatric hospital, geriatric unit, mild dementia patients (malnourished). ONS vs usual care & advice	I = 20 C = 15	Mild	AD	↑ Weight ↑ BMI	↓ Nutritional risk	→ Functional status → Cognitive function	3 weeks
Faxen-Irving 2002 Sweden [38]	CCT	Group-living for people with dementia. ONS & diet advice vs usual care	I = 21 C = 12	Mixed	Mixed	↑^a^ Weight ↑^a^ BMI ↑‡ TSF → AMC	N/A	→ ADL ↓ Cognitive function	5 months
FICSIT Fiatarone Singh 2000 USA [39, 86–88]	RCT	Nursing home (long term rehabilitation centre). ONS vs placebo	I = 24 C = 26	NR	NR	**↑** Weight **↑** BMI → MAMA → TBW	→ E intake → Fluid intake	→Functional status	10 weeks
FOPANU Carlsson 2011 Sweden [35, 89, 90]	RCT	Residential care facilities. Protein-enriched drink (+/− exercise) vs placebo drink (+/− exercise)	I = 96 C = 95	NR	NR	→ Weight → ICW	N/A	→ Balance → Gait → Lower limb strength	3 months
Gregorio 2003 Spain [40]	RCT	Nursing home residents with AD. Proprietary ONS (‘Nutrison’) vs usual food	IG = 24 C = 74	Mod	AD	↑* BMI	↓* Nutritional risk	N/A	12 months
Lauque 2000 France [44]	RCT	Nursing homes (risk of malnourishment). Proprietary ONS (Clinutren, Nestle) vs usual food	I = 19 C = 22	NR	NR	→ Weight → BMI	↑ E intake ↑ Protein intake	→ Grip strength	2 months
Lauque 2004 France [45]	RCT	Geriatric wards & day centres (risk of malnourishment). Proprietary ONS (Clinutren) vs usual food	I = 37 C = 43	Mod	NR	**↑** Weight **↑** BMI	↑ E intake **↓** Nutritional risk	→ADL → Cognitive function → Eating behaviour	3 months
Manders 2009 Netherlands [46]	RCT	Nursing homes. ONS vs placebo	I = 78 C = 33	NR	NR	**↑** Weight ↑ Calf circumference	N/A	→ Functional status → Grip strength → Cognitive status	24 weeks
Navrátilová 2007 Czech Republic [47]	RCT	Institutionalised residents with AD (type of institution unclear). Proprietary ONS (‘Nutridrink’) vs usual food	I = 50 C = 50	NR	AD	→ Weight → BMI	↑^†^ E intake ↑^†^ Protein intake	**↑** Cognitive function	1 year
Pivi 2011 Brazil [55]	RCT	Setting unclear. Proprietary supplement (Ensure with FOS®) vs usual care	I = 26 C = 27	Mild-severe	AD	↑* Weight ↑* BMI ↑* AMC ↑* AC → TSF	N/A	N/A	6 months
Planas 2004 Spain [48]	CCT (BA)*♣*	Dementia care day centre. ONS +/− micronutrients	I = 23 C = 21	Mild	AD	→ BMI ↑ MAMC ↑ TSF	↑ E intake	→ Cognitive function	6 months
Simmons 2010a USA [50]	RCT	Long-term care facilities, type unclear. Between meal nutritional supplements vs. usual care	I = 18 C = 20	NR	NR	→ Weight	→ E intake	↑ Costs & staff time	6 weeks
Souvenir I Scheltens 2010 USA & Europe [49, 91–96]	CCT (BA)*♣*	AD Treatment Centres. Proprietary ONS (Souvenaid) vs isocaloric placebo	I = 98 C = 97	Mild	AD	→ BMI	N/A	→ Cognitive function → QoL	12 & 24 weeks
Stange 2013 Germany [51, 97–99]	RCT	Nursing home (risk of malnutrition). ONS vs usual care	I = 45 C = 42	Mod-severe	NR	↑ Weight → BMI ↑ UAC ↑ Calf circumference	→ E intake → Protein intake → Nutritional risk	→ Cognitive function → ADL	12 weeks
Wouters-Wesseling 2002 Netherlands [53]	RCT	Nursing homes, residents with dementia. ONS vs placebo	I = 19 C = 16	NR	Mixed	↑ Weight → BMI	N/A	→ Functional status	12 weeks
Wouters-Wesseling 2006 Netherlands [52]	RCT	Psychogeriatric nursing homes (with acute infection). ONS vs usual care	I = 18 C = 16	NR	NR	→ Weight → TST → AMC	→ E intake	→ Functional status	5 weeks
Young 2004 Canada [54, 62, 100]	RCT	Dementia units within a nursing home. ONS vs high carbohydrate meals	I = 15 C = 19	NR	AD	↑ Weight	↑ E intake ↑ Protein intake	N/A	3 weeks
Fruit juice plus normal food vs control drink plus normal food									
Krikorian 2010a USA [42]	RCT	Community-dwelling. Grape juice vs placebo	I = 5 C = 7	MCI	N/A	→* Weight →* Waist	N/A	↑ Learning, → Spatial awareness, → Recall	12 weeks
Krikorian 2010b USA [43]	CCT	Community-dwelling. Blueberry juice vs placebo	I = 9 C = 7	MCI	N/A	→* Weight →* Waist	N/A	? Cognition, ? Spatial awareness	12 weeks
Krikorian 2012 USA [41]	RCT	Community-dwelling. Grape juice vs placebo	I = 10 C = 11	MCI	N/A	→ Weight → Waist	N/A	NR	16 weeks
Additional snacks between meals plus usual food vs usual food									
Simmons 2010b USA [50]	RCT	Long-term care facilities. Between meal snacks & assistance vs usual care	I = 25 C = 20	NR	NR	→ Weight	→ E intake	↑ Costs ↑ Staff time	6 weeks
Multicomponent interventions including ONS									
Beck 2010 Denmark [31, 101]	RCT	Elderly nursing home residents. ONS, Gratin diet, exercise, oral care vs usual care	I = 54 C = 55	NR	NR	↑ Weight ↑ BMI	→ E intake ↑ Protein intake	→ Cognitive performance → ADL	11 weeks
Boffelli 2004 Italy [33]	CCT (BA)	Malnourished residents of dementia unit. Individualised diet including mealtime assistance, environmental modification and ONS if required	19	Severe	Mixed	→ Weight → BMI ↑ Serum albumin	N/A	N/A	18 months

*Variance NR; ^†^significance stated but no *p* values presented; ^‡^Reported for females only; ^a^statistical significance reported in paper but change data not provided so significance does not appear in meta-analysis, ♣ these were RCTs, but we used their data as before-after comparisons, so they are reported here as BA.

↑ indicates statistically significant increase; ↓ indicates statistically significant reduction; → indicates no statistically significant effect; statistical significance of all effects were checked by reviewers where data were available, and reviewers results used when they differed from the original paper.

*AD* Alzheimer’s disease, *AC* Arm Circumference, *ADL* activities of daily living, *AMC* arm muscle circumference, *BA* before-after or pre-post, *BMI* body mass index, *C* control group, *CCT* controlled clinical trial (with a concurrent control arm unless indicated as BA), *CDR* Clinical Dementia Rating Scale, *C* control group, *E* energy, *GDS* Global Deterioration Scale, *I* intervention group, *ICW* intracellular water, *MAMA* mid-arm muscle area, *MAMC* mid-arm muscle circumference, *MCI* mild cognitive impairment, *mod* moderate, *N/A* not applicable, *NR* not reported, *ONS* oral nutritional supplement, *QoL* quality of life, *RCT* randomised controlled trial, *suppl* supplement, *TSF* triceps skin fold, *TST* triceps skin fold thickness, *TBW* total body water, *UAC* upper arm circumference, *vs* versus

**Table 3:** Characteristics and results of included food and drink modification, swallowing intervention, eating or drinking assistance and social support interventions

Study	Design	Setting, Intervention type	No.	Dementia stage	Dementia type	Effect on Nutrition / hydration status	Intake effect	Quality & other outcomes	Duration
Swallowing interventions									
Bautmans 2008 Belgium [56]	RCT	Nursing home. Cervical spine mobilization to help dysphagia	15	Severe	AD	NR	NR	↑ Dysphagia limit	1 week
Germain 2006 Canada [57]	RCT	Long term care facility. Dysphagia diet	I = 8 C = 9	NR	AD & others	↑ Weight	↑ E intake	NR	12 weeks
Robbins 2008 USA [58, 102–104]	RCT	Hospitals & nursing homes. 1. Nectar-thick or 2. Honey-thick consistency fluids 3. Chin-tuck position	Nectar 133, Honey 123, Chin-tuck 259	Various	NR		NR	→ Aspiration pneumonia incidence (for thickened vs chin-tuck)	3 months
Food modification									
Beck 2010 Denmark [31, 101]	RCT	Elderly nursing home residents. ONS, Gratin diet, swallowing problem management, exercise and oral care vs usual care	I = 54 C = 55	NR	NR	**↑** BMI ↑ Weight	→ E-intake ↑ Protein intake	→ Cognitive performance → ADL	11 weeks
Boffelli 2004 Italy [33]	CCT (BA)	Dementia unit. Diet & environment modification, feeding assistance and supplements	29	Severe	Various	→ BMI → weight ↑ Albumin	NR	NR	18 months
Jean 1997 USA [59]	CCT (BA)	Nursing home. Finger food menu	12	NR	AD & others	? Weight loss arrest	NR	? Eating independence	6 months
Keller 2003 Canada [63, 105]	CCT	Long term care facilities. Individualised food service, food modification, education and dietitian time	I = 33 C = 49	NR	AD & others	↑ weight	NR	NR	21 months
Kenkmann 2010 UK [64, 106]	RCT	6 Care homes. Dining environment & menu changes	I = 57, C = 48	NR	NR	→ Weight, → BMI, → Hydrated	NR	→ Enjoyment of food/drink	1 year
Salas-Salvado 2005 Spain [61]	RCT	Geriatric institutions. Meal replacement with commercial lyophilised supplement	I = 15 C = 23	Severe	AD	↑ Weight ↑ Serum albumin	→ E intake → Nutritional risk	→ Eating behaviour → Mortality → Cognitive parameters	3 months
Soltesz 1995 USA [60]	CCT (BA)	Alzheimer’s Care Centre. Finger food provision	43	NR	AD	→ Weight	↑ Proportion food eaten	NR	6 months
Young 2005 Canada [54, 62, 100]	RCT	Nursing home. High CHO dinners	I = 15 C 19	NR	AD	NR	↑ E intake	NR	21 days
Eating or drinking assistance interventions									
Boffelli 2004 Italy [33]	CCT (BA)	Dementia unit, diet & environment modification, feeding assistance and supplements	29	Severe	Various	→ BMI → Weight ↑ Albumin	NR	NR	18 months
Simmons 2001 USA [65, 107]	CCT	Nursing Homes. Staff assistance, prompting, food/drink service and exercise	I = 48 C = 15	NR	NR	→ Serum osmolality → BUN: creatinine ratio	→ Food & fluid intake	NR	32 weeks
Simmons 2008 USA [66]	RCT	Skilled nursing homes. Either meal time or between meal feeding assistance	I = 35 C = 34	NR	NR	**?** BMI, ? Weight	**↑** E intake	NR	24 weeks
Simmons 2010a USA [50]	RCT	Long-term care facilities. Between meal supplements & assistance vs usual care	I1 = 18 C 20	NR	NR	→ Weight	→ E intake	NR	6 weeks
Simmons 2010b USA [50]	RCT	Long-term care facilities. Between meal snacks & assistance vs usual care	I2 = 25 C = 20	NR	NR	→Weight	→ E intake	NR	6 weeks
Wong 2008 New Zealand [67]	CCT (BA)	Short stay assessment unit.Individual mealtime assistance	7	NR	NR	**↑** BMI	**↑** E intake	NR	12 weeks
Studies with a strong social element around eating/drinking									
Altus 2002 USA [68]	CCT (BA)	Locked dementia unit. Family-style meals −/+ staff training	5	Mod- severe	AD & others	NR	NR	? Resident Participation in mealtime tasks ? Appropriate communication ? Praise statements ? Staff satisfaction with resident participation	5 days each period
Charras 2010 France [69]	CCT	Dementia units in nursing homes. Shared mealtime with staff	I = 8 C = 10	Severe	AD	**↑** Weight	NR	? Autonomy ? Quality of interactions ? Food quality	6 months
Huang 2009 Taiwan [70]	CCT (BA)	Older person care facility, Reminiscence cooking therapy	12	Mild-mod	NR	NR	NR	→ MMSE ↑ Happiness → Communication ? Participation	8 weeks
Santo Pietro 1998 USA [71]	CCT	Dementia unit within a nursing home. Breakfast club (communication therapy)	I = 20 C = 20	Mild-mod	AD	NR	NR	↑ Interest & involvement ↑ Communication	12 weeks

↑ indicates statistically significant increase; ↓ indicates statistically significant reduction; → indicates no statistically significant effect; ? indicates unclear whether effect was statistically significant. Statistical significance of all effects were checked by reviewers where data were available, and reviewers results used when they differed from the original paper.

*AD* Alzheimer’s disease, *ADL* activities of daily living, *BA* before-after, *BMI* body mass index, *CCT* controlled clinical trial, *CG* control group, *CHO* Carbohydrate, *E* energy, *IG* intervention group, *NR* not reported, *ONS* oral nutritional supplement, *RCT* randomised controlled trial, *vs* versus

### Oral nutrition supplement (ONS) interventions

#### Effects of ONS

Twenty seven interventions (including approximately 1300 participants) [30–55] investigated effects of ONS. We defined these as studies where any oral food or drink was offered in addition to standard provision (including commercial ONS preparations and additional snacks/drinks). In 19 interventions the supplement was the only intervention and compared to usual care or placebo, whilst in two it was part of a multicomponent intervention [31, 33] and in four was compared to another intervention [48, 50, 54, 55]. Twenty studies were RCTs and six, CCTs. Study duration was from three to 80 weeks.

Few studies demonstrated low risk of selection bias, performance bias, detection bias or funding bias, and no studies were at low risk of bias overall (Additional file 3: Figure S3.1). Risk of attrition bias was low in over half of included studies. Due to lack of pre-registered protocols and details, most studies were judged at unclear risk of reporting and/or selection bias. Comparability of participants at baseline, suitability of outcomes measured and dementia diagnosis appeared to be satisfactory in most studies.

### Effects of interventions for swallowing problems

#### Supplementary fruit juice

**Fig. 2:**
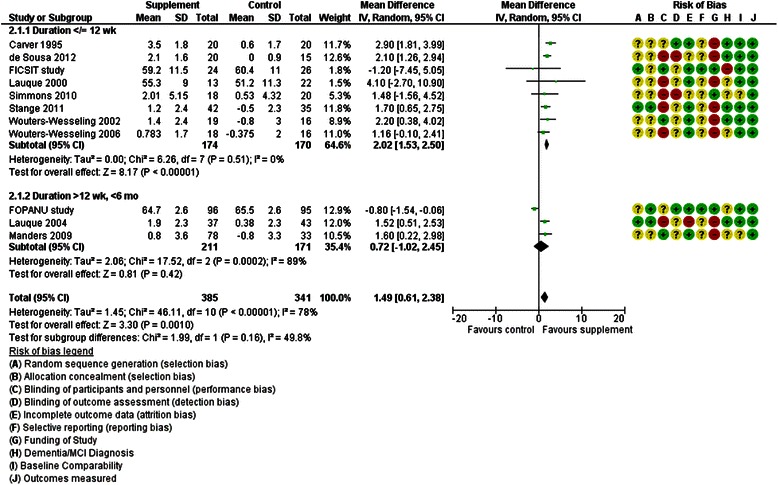
Forest plot of the effect of RCTs of ONS plus usual food vs usual food alone on weight (in kg). * de Sousa 2012 [37], Simmons 2010 [50], Stange 2011 [51], Wouter-Wesseling 2002 [53] and 2006 [52] provided change data

**Fig. 3:**
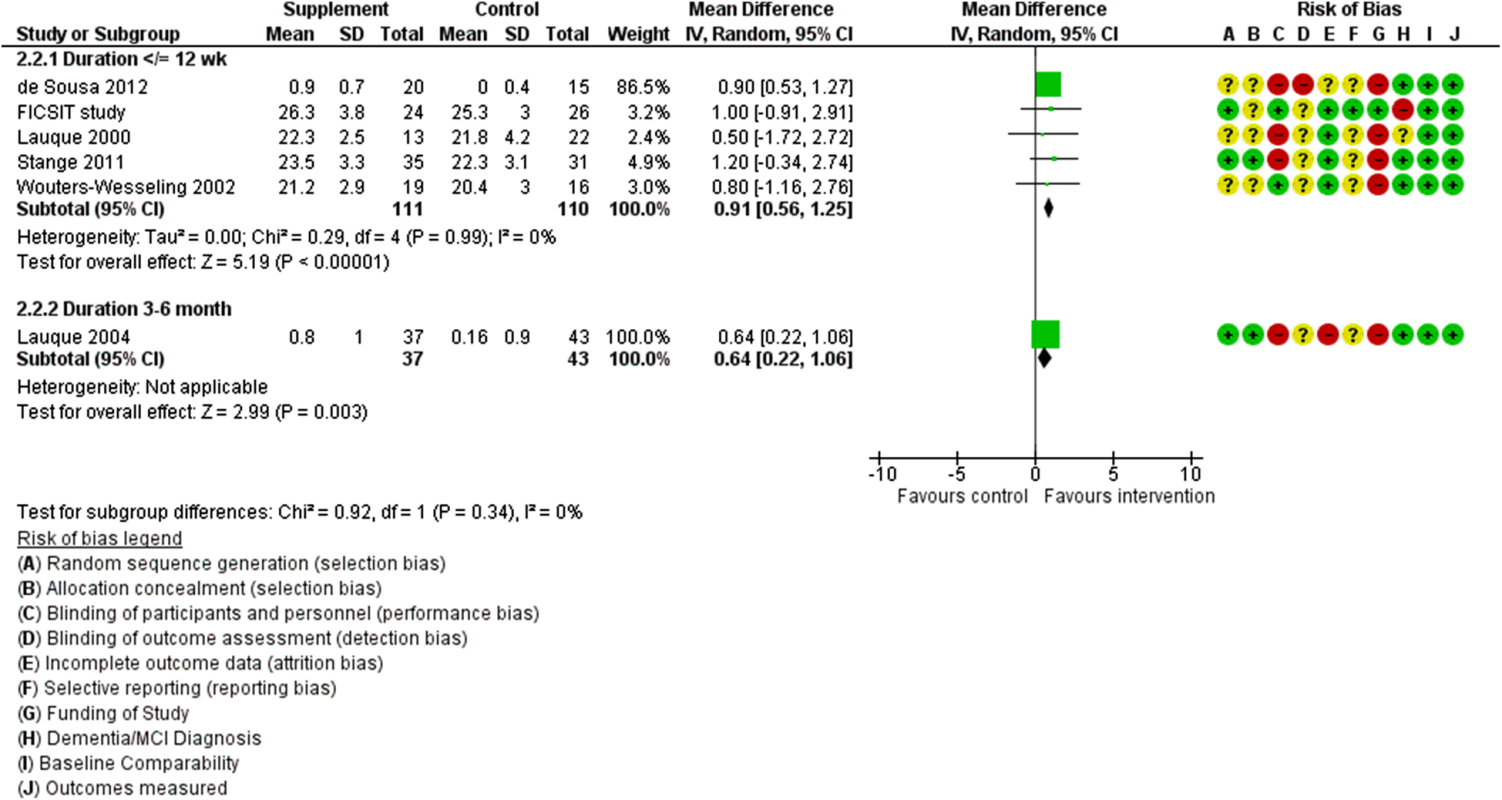
Forest plot of the effect of RCTs of ONS plus usual food vs usual food alone on body mass index (in kg/m^2^)

These studies assessed the effects of providing additional energy and protein using ONS. Of 17 RCTs included, 11 assessed the effects of ONS plus usual food vs usual food alone on weight or body mass index (BMI), and reported results that could be incorporated into meta-analysis. Duration of three was >12 weeks and meta-analysis suggested no statistically significant effect on weight (mean difference [MD] 0.72 kg, 95 % CI −1.02-2.45, 382 participants) but with high heterogeneity (I^2^ 89 %) (Fig. 2). One study [45] also assessed effects on BMI, suggesting a small, statistically significant, increase of 0.64 kg/m^2^ (95 % CI 0.22-1.06, 80 participants) (Fig. 3).

Eight RCTs assessed effects over 3–12 weeks. Meta-analysis suggested small, statistically significant, effects of supplementation on weight (2.02 kg, 95 % CI 1.53-2.50, 344 participants, I^2^ 0 %, Fig. 2) and BMI (0.91 kg/m^2^, 95 % CI 0.56-1.25, I^2^ 0 %, 221 participants, Fig. 3). Six RCTs either did not report weight or BMI [30], suggested no effect [32, 40, 47], or reported improvements but presented data in a form that could not be included in meta-analysis [48, 54]. We did not include CCT results in meta-analyses as they provide less valid data on effects. One CCT [38] reported significant weight gain (3.5 kg) compared to control after 5 months ONS (p = 0.003) while two RCTs [48, 49] (analysed as before-after [BA] assessing effects of ONS compared to baseline) suggested no effect at six months on BMI.

Effects on other anthropometric measures were mixed: One RCT [36] reported positive effect on Mid-upper Arm Muscle Circumference but not on Triceps Skinfold Thickness; in three RCTs [46, 51, 55] Arm Muscle Circumference and/or calf circumference improved significantly; in a small Dutch RCT [52] there was no effect on Triceps Skinfold Thickness or Arm Muscle Circumference. One CCT [48] reported significantly increased Triceps Skinfold Thickness and Mid-Upper Arm Circumference.

Two RCTs [37, 45] assessed effects on Mini-Nutrition Assessment (MNA), and meta-analysis suggested significant improvement with supplementation (1.41 points, 95 % CI 1.06-1.75, I^2^0 %, 115 participants, see Additional file 3: Figure S3.2). Meta-analysis of six RCTs [39, 44, 45, 50–52] suggested small statistically significant increases in energy intake with ONS of 132 kcal/day (95 % CI 41–223, 279 participants, I^2^14 %, Additional file 3: Figure S3.3).

Quality of life was measured in one RCT [51] with no significant effect on any of nine sub-scales for quality of life, except ‘positive self-perception’ (*p* = 0.011), and no effect on quality of life was found in the single CCT [49]. Functional status was assessed in several RCTs and CCTs but none found any effect (Table 2). Cognitive status was assessed in five RCTs and three CCTs. Three RCTs and all the CCTs found no effect [37, 38, 45, 48, 49, 51], while one RCT [30] reported an improvement of 6 points in the MMSE score (*p* < 0.001, a surprising finding) and another [47] reported slowing the rate of cognitive decline (−1.3 vs. -3.7, *p* = 0.024). Hydration status was measured in one RCT [39] which reported no effect.

Five RCTs [35, 37, 39, 40, 53] reported 40 deaths in 419 participants, but meta-analysis did not suggest any effect of supplementation on mortality (RR 0.83, 95 % CI 0.43-1.61, Additional file 3: Figure S3.4).

### Effects of food and drink modification

#### Additional snacks between meals

Three studies assessed effects of fruit juice plus normal food vs control drink plus normal food. Two small RCTs [41, 42] examined effects of daily grape juice vs placebo for 12 or 16 weeks in community dwelling older adults with MCI, finding no significant effect on weight or waist circumference. One CCT [43] found no effect of 12 weeks of blueberry juice vs placebo in nine adults with MCI on weight, but suggested improvement of some memory tests (no clear numerical data presented, and control data utilised from another study) [42].

### Effects of eating and drinking assistance

#### Multicomponent interventions including ONS

A US RCT investigating energy intake and cost effectiveness of between-meal nutritious snacks in 45 nursing home residents [50] found no significant effect at five weeks on weight (*p* = 0.66), or energy intake (*p* = 0.20). Staff time increased (by 1.7 minutes/resident/offer), as did product and staff costs (*p* < 0.01). A CCT [34] on 15 people living in Swedish dementia facilities reported significant weight *loss* after six months drinkable probiotic yogurt (−3.9 kg, SD 3.9, range −9.2-3), but no change in energy or fluid intake.

### Effects of interventions with a strong social element around eating and drinking

One RCT [31] tested effects of 11 weeks of ONS, management of swallowing problems with soft pureed gratin diet, oral care and exercise vs usual care in 121 Danish dementia nursing home residents, finding improved weight (1.3 % vs. -0.6 %, p = 0.005) and BMI (0.4 % vs. -0.2 %, *p* = 0.003). Energy intake (0.7 vs.-0.3 MJ/day, *p* = 0.084), functional and cognitive status did not alter. A CCT [35] evaluated diet and environmental modification, increased nurse time and assistance at meals in 19 malnourished Italian dementia unit residents, finding increased serum albumin (3.0 to 3.4 g/dL at 18 months) but no changes in weight or BMI.

### Effects of interventions for swallowing problems

Swallowing interventions included interventions aiming to assess, treat or manage swallowing problems. Three RCTs [56–58] assessed effectiveness of interventions to support people with swallowing problems, two had low risk of selection bias [57, 58], all three had high or unclear risk of performance bias and one [56] was at low risk of detection bias (Additional file 3: Figure S3.5). None was at low risk of bias overall, and meta-analysis was not possible due to differences in interventions.

One RCT [57] examined effectiveness of a dysphagia diet (reformed minced/pureéd foods and thickened fluids) vs standard diet in 17 cognitively impaired residents with weight loss and swallowing problems (their stage of dementia was not defined). Weight (+3.9 kg vs. -0.79 kg, *p* = 0.02) and energy intake increased (*p* = 0.03). The second RCT [58] compared thickened liquids vs chin-down drinking in aspirating patients (nutritional status and dementia stage were not defined) finding no differences in aspiration pneumonia incidence or death (with no control group, it is unclear whether interventions were effective overall).

The third RCT [56] assessed one week cervical-spine mobilization vs therapist socialising visits in 15 people with severe AD and dysphagia. Dysphagia limit (maximal bolus of water swallowed in a single movement) increased from 3 to 10 ml (median), significantly greater than control (*p* = 0.03), but fluid intake and hydration status were not measured.

### Effects of food and drink modification

Food and drink modification included any modification to the food/drink itself, including nutritional or textural alterations and finger foods (except specifically for those with swallowing problems). All eight interventions were at high or unclear risk of selection, performance and detection biases, so none were at low risk of bias overall (Additional file 3: Figure S3.6. Meta-analyses were not possible due to differing interventions and outcomes.

### Finger foods

Two CCTs [59, 60] assessed use of finger foods. One [59] evaluated six months of finger food menu for 12 cognitively impaired residents with poor intake and limited use of cutlery, finding weight-loss arrested in 10/12 participants and eating independence improved (though no numbers or statistical analysis were provided). The other [60] assessed effects of increased finger food provision on weight and food consumption of 43 US AD care centre residents. The intervention, though aiming to increase numbers of finger foods provided, had implementation problems in that numbers of finger foods provided increased minimally (2.57 to 2.71 [breakfast]; 2.19 to 2.38 [lunch] and 2.0 to 3.8 [dinner]) over the six month study period. Body weight did not increase, though the proportion of food eaten increased by 3 % (*p* < 0.05).

### Other food modification

Two RCTs [61, 62] assessed effects of food modification. Three months meal replacement with lyophilised (freeze-dried) foods and advice to residents and carers vs advice alone was investigated in 53 people with severe cognitive impairment [61], reporting improved weight (+2.06 kg vs +0.32 kg, *p* < 0.05) and serum albumin (3.76 mg/dl vs 1.13 mg/dl, *p* < 0.05), but no differences in MNA, eating behaviour, cognition or mortality. A crossover RCT [62] assessed 21 days high-carbohydrate dinners compared to usual intake in 34 nursing home residents with AD. Energy intake increased, but weight was not presented for the first part of the crossover, so could not be assessed.

### Food modification as part of multi-component interventions

One RCT [31] (discussed under multicomponent ONS interventions) and three CCTs [33, 63, 64] assessed food modification as part of multi-component interventions: One CCT [33] found increased serum albumin (3.0 to 3.4 g/dL) at 18 months in malnourished people with severe dementia living on a dementia unit, without effects on weight or BMI; the second [63] reported significant weight gain after nine months of enhanced dietetic time, high-energy snacks, high-energy/protein mashed potatoes, high-protein milk and blended enhanced breakfasts (4.8 % vs. -4.5 %, *p* < 0.001) in orally fed people with dementia (dementia stage not stated); and the third [64] found that increased choice, ability to self-serve, improved atmosphere and readily available drinks and snacks had no effect on nutritional status, hydration or enjoyment of food in care home residents with low mean MMSE (not selected for cognitive or nutritional status).

### Effects of eating and drinking assistance

Eating and drinking assistance was defined as direct physical assistance provided by carers, staff or volunteers to enable eating and/or drinking including use of aids and extra staff, but not verbal prompting. Six interventions [33, 50, 65–67] assessed effects of eating or drinking assistance, of which all were judged at high or unclear risk of selection and performance bias, while one was at low risk of detection bias, and none were at low risk of bias overall (Additional file 3: Figure S3.7). Meta-analysis was not possible due to differing interventions and outcomes. One RCT and one CCT investigated effects of eating assistance on nutritional status of people with dementia [66, 67]. A US study [66] randomised nursing home residents to individual assistance at mealtimes or between-meals vs usual care. Energy intake was higher with assistance (*p* = 0.03), but BMI and weight (though measured) were not reported at 24 weeks. Staff time at 48 weeks was >42 minutes/meal/resident, and 14 minutes/snack/resident, and was <10 minutes/meal/resident in the control group at 24 weeks. A CCT [67] (period 3) used volunteers to assist patients during mealtimes, reporting increased BMI (+0.4 kg/m^2^, *p* < 0.04) and energy intake (+44Kcal, *p* < 0.001) after 12 weeks (though it is surprising that such small changes were statistically significant in seven participants).

Four interventions [33, 50, 65] had eating assistance as part of a multicomponent intervention: A CCT [33] (discussed above) included mealtime assistance, finding increased serum albumin at 18 months, without effects on weight or BMI; a complex 32-week CCT (including exercise, toileting and drinking prompts) [65] found no change in meal-time food or fluid intake, or hydration status; an RCT [50] provided assistance to enhance eating intake and independence alongside between-meal nutritional supplements or nutritious snacks compared to usual care, finding no effect on weight or energy intake at five weeks, but with increased staff time and costs.

### Effects of interventions with a strong social element around eating and drinking

Of four CCTs [68–71] which studied the promotion of eating and/or drinking alongside a social activity or within a social context, all were at high or unclear risk of selection and performance bias, and one was at low risk of detection bias (Additional file 3: Figure S3.8). None was at low risk of bias overall.

Family-style meals in five US moderate-severe dementia unit residents were associated with increased resident participation in mealtime tasks, appropriate communication, frequency of praise and carer satisfaction with residents’ participation levels, but statistical significance was unclear [68]. Another study of family-style meals taken with staff [69] reported increased weight (+3.4 kg vs −2.2 kg, *p* = 0.02) in18 patients with severe AD, while comments and observations suggested improvements in autonomy, quality of interactions and other aspects of enjoyment of meals by patients. A study of reminiscence cooking therapy in 12 Taiwanese mild-moderate dementia nursing home residents improved most personal interaction scale items including feeling of happiness (*p* = 0.01) and positive communication (*p* = 0.05) [70]. A facilitated breakfast club compared to discussion with coffee in 40 people with mild-moderate AD in a dementia unit [71] reported significantly higher scores for interest, involvement and procedural memory, significantly better functional and cognition status.

### Answers to questions from lay stakeholders

We found limited data to answer the questions formulated through public involvement (Table 1).

## Discussion

To our knowledge, this is the first systematic review to assess the effectiveness of a full range of direct interventions aiming to improve, maintain or facilitate eating and drinking in adults with dementia of any type, any degree and in any setting. The 43 interventions investigated the effect of oral supplements, food/drink modification (including their use for people with swallowing problems), eating assistance and social support. Most interventions occurred within residential institutions of various types, but three interventions on people with MCI were in the community [41–43], one a treatment centre (assumed to be an outpatient setting [72]), one in day centres [48] and one unclear [55]. Included studies were mostly small (from five to 515 participants, with only seven having ≥100 participants [31, 35, 46, 47, 58, 64, 73] and none were at low risk of bias.

Meta-analysis of RCTs of ONS suggest small, short term, statistically significant effects (of ONS with usual food compared to usual food alone) on weight, BMI, MNA and energy intake, but effects were too small to be clinically useful, and it is unclear whether these effects were sustained in the longer term (Figs. 2 and 3). There were no clear effects on the relevant and important outcomes quality of life, functional or cognitive status, hydration status or mortality, nor on fluid intake, and data on more useful nutritional outcomes (such as albumin or iron status markers) were rare. There was insufficient evidence to assess effects of fruit juice supplements, nutrients without additional energy and protein or snacks between meals.

We found limited evidence that, for those with swallowing problems, dysphagia diets improved some nutritional markers [57]. Evidence for the utility of cervical spine mobilisation [56], different consistencies of thickened drinks, and chin-down drinking [58], were less clear. Effects of food modification were also mixed. In those unable to use cutlery, there was little evidence of finger food utility, with one study showing problems in implementation [60] and the other suggesting positive effects but with no numerical data provided [59]. In those with severe dementia there was some evidence that liquid or semi-liquid foods improved weight a little over 3 months (but not eating behaviour) [61]. Multicomponent interventions including food modification increased weight and BMI in some cases [31, 63] but not others [33, 64].

Studies assessing effects of assistance with eating and drinking suggested little effect on nutritional outcomes. Studies assessing social interventions were small but consistently suggested the possibility of improving important aspects of quality of life including autonomy, communication, mood, involvement and participation in meaningful activity in the context of food and drink [68–71].

As this systematic review did not find sufficient high quality evidence to state that any particular intervention was clearly effective, or clearly ineffective, we suggest that these interventions require further research. High quality, well designed randomised trials should be tailored specifically for people with dementia at different stages of the disease and in different settings. They should include robust outcomes such as weight change to clarify nutritional status, serum osmolality to inform on hydration status and health outcomes (several studies reported improvements in intake, without accompanying improvements in nutritional status indicating that intake measures should be interpreted cautiously). Trials will ideally include input from people with dementia, and their formal and informal carers in study design. Trials should be powered to produce useful data and consider longer term effects (over at least 6 months) of interventions.

Despite no clear evidence of effectiveness, people with dementia and their carers regularly have to deal with problems around eating and drinking, including needing to be fed, food refusal, and swallowing difficulties [7]. For this reason, they may like to read about the different interventions that have been tested, and choose one or more of the interventions to help solve their own problems. The possible interventions are detailed in Table 4.

**Table 4:** Promising interventions that are presently unproven, but that warrant early reassessment in high quality and well powered RCTs^a^

Aim	Potential interventions (presently unproven) which warrant early reassessment
Increase weight and/or BMI	o Oral Nutrition Supplements (ONS) (Figs. 2 and 3) o ONS, gratin diet for those with swallowing problems, plus exercise and oral care (Beck) [31] o Dysphagia diet (reformed minced and pureed foods and thickened fluids) for those with swallowing problems (Germain) [57] o Meal replacement with commercial lyophilised supplement (Salas-Salvado) [61] o Multifactorial intervention including enhanced menu, individualised food service, more dietetic time, increased nutritional awareness and communication (Keller) [63] o Individual mealtime assistance (Wong) [67] o Shared mealtime with staff (Charras) [69]
Improve hydration	o No particularly useful interventions were noted, but cervical spine manipulation appeared to increase dysphagia limit for those with swallowing problems (Bautmans) [56]
Support meaningful engagement with food and/or drink	o Eating with carers (Charras) [69] o Family style meals for people with dementia, enhanced further by staff training (Altus) [68] o Facilitated breakfast club with supported involvement in preparing, conversing, eating and clearing up (Santo Pietro) [71]
Improve quality of life	o Reminiscence cooking sessions (Huang 2009) [70]
Support eating independence	o No particularly useful interventions assessed
Improve quantity, quality or adequacy of food or fluid intake	o Combination of ONS, gratin diet, exercise and oral care (Beck) [32] o Finger food provision (Soltesz) [60] o High carbohydrate dinners (Young) [62] o Meal time or between meal feeding assistance, or individual mealtime assistance (Simmons 2008, Wong 2008) [66, 67] o Dysphagia diet (reformed minced and pureed foods and thickened fluids) for those with swallowing problems (Germain) [57]

^a^If you or someone you care for is experiencing difficulties with eating or drinking ALWAYS discuss these eating and drinking problems with your/their doctor, and ask to be referred to a dietitian and/or Speech and Language Therapist

While previous systematic reviews have assessed potential nutritional causes and prevention of dementia [74–76], literature examining the effectiveness of interventions to support people with dementia to eat and drink well is more limited. Systematic reviews exploring effectiveness of interventions to support eating and drinking in people with dementia were either limited to residential care settings [23–25], focused on limited interventions or outcomes [26], or carried out using limited or ill-described searches [27, 28], hence leaving evidence gaps.

### Strengths and limitations

A strength of this systematic review is that after an extensive search we found a comprehensive set of interventions aiming to improve, maintain or facilitate eating and drinking in people with dementia and MCI. All study selection and data extraction was conducted in duplicate and we carried out meta-analyses where studies were appropriately similar in intervention, comparison and outcome, but this was only feasible for RCTs of ONS. The scope was enhanced by lay stakeholder input to ensure we investigated relevant objectives. A limitation of our review may be that despite the extensive search, some studies might have been missed due to poor indexing and abstracts omitting to identify participants as having dementia or cognitive impairment. To overcome this, we included studies where cognitive scores suggested that most participants were cognitively impaired even when dementia was not mentioned or formally diagnosed.

We assessed interventions for swallowing problems in people with cognitive limitations, and found very limited evidence of the utility of interventions. However, whilst swallowing problems are common in people with dementia it is likely that results of research on interventions for people with swallowing problems but without dementia are transferrable to people with dementia, so the evidence base is much larger. Recent systematic reviews on interventions for people with swallowing problems provide a fuller data set [77, 78].

As included studies often lacked details of dementia diagnosis, type and/or staging it was difficult to assess what interventions may be useful in different types and stages of dementia. There is also a lack of clear data on nutritional and hydration status of participants, and studies not choosing people with specific nutritional deficits may limit our ability to see effects of interventions on nutritional outcomes [79]. Eating and drinking problems differ at different dementia stages, so it is likely that interventions will be stage- or problem-specific. Positive effects of nutritional supplements have been reported by other reviews [80, 81] but these reviews did not consider duration. Previous reviews have not considered meaningful activity or quality of life as primary outcomes, despite significantly positive relationships between psychological wellbeing and nutritional status in dementia [80–82]. Outcomes such as satisfaction, autonomy and quality of life have been highlighted in an expert-led research agenda as important determinants of interventions to improve food and fluid intake for older people, but were reported in few included studies [83]. The wide range of interventions and variable outcome measures reported make it difficult to compare results between studies.

Although we could not report definitive evidence on effectiveness of one or more interventions, by reviewing all available studies we have presented evidence on potentially useful interventions that could (and should) be considered for further research. We present a list of potentially useful interventions that need to be assessed in high quality and well powered RCTs to properly assess their efficacy (Table 4), and this may be a useful source of inspiration for people with dementia and their carers as suggestions to try out in dealing with specific eating and drinking problems, even though they have not been shown to be clearly effective. However, it should be noted that potentially effective interventions may not have appeared effective in this systematic review because many of the studies were underpowered, and so unable to suggest statistically significant benefits or harms.

Our finding that strong social support around food and drink are positive for quality of life is supported by qualitative research in people with dementia that “eating together reveals the essence of what it is to be human” and mealtimes reflect identity and connections [84] and anthropological research suggesting that eating together is a core human activity important to building social groups [85]. This is a priority for the research agenda.

## Conclusions

While we found no definitive evidence on effectiveness, or lack of effectiveness, of specific interventions, studies were small and short term. As people with cognitive impairment and their carers have to tackle eating problems despite this lack of evidence, promising interventions have been listed and include: Oral nutrition supplements; pureed and reformed foods; thickened fluids; individual mealtime or between-meal assistance; family style meals and meals shared with staff or carers; meals with a facilitated social element; reminiscence cooking; finger food provision. The need remains for high quality trials tailored for people with progressive cognitive impairment and their carers assessing robust and relevant outcomes.

## Additional files

Additional file 1:
**Full systematic review methodology for EDWINA (Eating and Drinking Well IN dementiA) systematic review.** (DOCX 31 kb) Additional file 2:
**Detailed characteristics of direct intervention EDWINA (Eating and Drinking Well IN dementiA) included studies.** (DOCX 125 kb) Additional file 3:
**Summary of study validity and further meta-analysis results of direct interventions.** (DOCX 136 kb)

## Abbreviations

CCT: non-randomised controlled trial
EDWINA: Eating and Drinking Well IN dementiA study
MCI: mild cognitive impairment
ONS: oral nutrition supplements
RCT: randomised controlled trial
