# Supplementary material for: Effectiveness of interventions to directly support food and drink intake in people with dementia: systematic review and meta-analysis
Source: BMC Geriatr. 2016 Jan 22;16:26. doi: 10.1186/s12877-016-0196-3 (PMC4722767; doi:10.1186/s12877-016-0196-3)
Supplement: Additional file 2: — Detailed characteristics of direct intervention EDWINA (Eating and Drinking Well IN dementiA) included studies. (DOCX 125 kb) [file 12877_2016_196_MOESM2_ESM.docx]

**Supplementary File 2: Detailed characteristics of direct intervention EDWINA (Eating and Drinking Well IN dementiA) included studies**

| **Study** | **Participants** | **Interventions** | **Nutrition/hydration status outcomes** | **Nutrient/ fluid intake outcomes** | **Quality & other outcomes** |
| --- | --- | --- | --- | --- | --- |
| **Oral supplementation studies** | | | | | |
| **Abalan 1992 (1)**  **France**  **Setting:** Geriatric inpatients  **Aim**  To examine the effects of total oral nutritional supplementation on cognitive function in SDAT | **Participants**: AD inpatients  **N:** 29 (15 int, 14 Cont)  **M/F:** 1/28  **Mean age (SD)**: *Int* 85.13 (7.78), *Cont* 85.36(6.28) *85.24(6.97)*  **Nutritional status:**  Weight, Kg, mean (SD): *Int* 48.9 (9.2), *Cont* 46.3(10.7) *47.64(9.86)*  **Cognitive status: MMSE, mean score (SD)** *Int* 11.47 (3.93), *Cont* 9.71(4.6) *9.92(4.26)*  **Dementia** **diagnosis:** NINCDS-ADRDA criteria  **Dementia type:** AD  **Dementia stage:** NR  **Acute illness:** NR | **Design:** RCT (parallel)  **Int: Type:** ONS  **Provided by:** Nursing staff (probably)  **Details:** 2x 200 kcal cartons of Tonexis & Tonexis HP (increased to 4x200 kcal at day36) plus Ca, Phosphorus, Mg and trace elements and normal hospital food  **Cont:** normal hospital food  **Duration of Int/ follow up:** 105 days  **Compliance:** NR  **Individualised:** No  **Ethics obtained:** NR | **NR** | **E intake, kcal/day, mean (SD):** (for only 9 participants, 5 int & 4 Cont) No baseline but period 1 (days 1-35) & 2 (days 36-105)  P1: int. 1254 (259) + 400 Kcal ONS vs Cont 1386 (440)  P=0.6  P2: int 936 (235) + 800 Kcal ONS. vs Cont 1386 (440)  P=0.06 | **Change in MMSE, mean (SD):**  int +3.47 (2.1) vs Cont -2.71 (4.29)  P<0.0001 |
| **Beck 2002 (2)**  **Denmark**  **Setting**  **Nursing home**  **Aim:**  To examine the effects of a home-made oral supplement on body weight and energy intake of old people residing in nursing home | **Participants**: NH residents at risk of malnutrition (MNA 17-23.5)  **N:** 16 (Int 8, Cont 8)  **M/F:** 6/10  **Mean age (range)**: int 84 (65-96)  Cont 87 (77-91)  **Nutritional status:** BMI, mean (range) int, 20 (15-22), Cont 21 (14-24)  **Cognitive status:** CPS, mean (range) 4 (3-5)  **Dementia diagnosis:** NR  **Dementia type:** NR  **Dementia stage:** NR  **Acute illness:** NR | **Design: RCT (parallel, gps B&C)**  **Int**: Type: home-made oral supplement  **Provided by: care home staff?**  Details: Homemade oral supplement given every evening for 2 months. One serving= 2 dL (384 kcal, 73% fat & 5% protein)  **Cont**: normal food  **Compliance:** median intake 1.57 MJ/d. described as high compliance  **Duration of Int/ follow up**: 2 months  **Compliance:** Median intake 1.57 MJ/day (95%CI 0.15-1.6)  **Individualised:** No  **Ethics obtained:** Yes | **Change in body weight, kg, median (95% CI)**  Int. +1.3 (-1.0-3.0)  Cont +1.5 (-2.3-9.0) | **Change in energy intake, MJ/d, median (95% CI)**  Int -0.1 (-1.9-3.6)  Cont 0.1 (-0.7-2.0) |  |
| **Beck 2010 (28)**  **Denmark**  **Setting**  **Nursing home**  **Aim:**  To test the hypothesis that a multifaceted 11 week intervention would have a significant influence on nutrition and function in elderly nursing home residents | **Participants:** elderly NH residents  **N:** 121 randomised (int 62, Cont 59), 109 analysed (int 54, Cont 55)  **M/F:** 33/88  **Age, median (95% CI): Int** 87 (84-90), Cont 86 (84-87)  **Nutritional status: BMI , median (95% CI):** int 23.4 (21.8-24.8), Cont 23.4 (21.3-25.2)  **Cognitive status: CPS, median (95% CI):** int 3 (2-5), Cont 3 (2-3)  **Dementia diagnosis:** NR  **Dementia type:** NR  **Dementia stage:** NR  **Acute illness:** NR | **Design:** RCT (parallel)  **Int: Type:** Multifaceted (nutrition, exercise & oral care)  **Provided by:** Care home staff & health workers  **Details: Nutrition:** 25g chocolate + 150ml hot chocolate drink or home-made supplement/ day + 150 ml oral training suppl provided after exercise twice/week. Gratin diet for people with swallowing difficulties.  **Exercise:** 40-60 min moderate intensity individualised exercise, groups of 2-5, 2 sessions/week supervised by physiotherapists.  **Oral care:** dental hygienist 1-2/week to reduce plaque prevalence.  **Cont:** Usual care  **Duration of Int/ follow up:** 11 weeks/ 27 weeks  **Compliance:** NR, although state excluded non-compliant participants.  **Individualised:** Yes  **Ethics obtained:** Yes | **% Change in BMI, median (95% CI)**  **Int: 0.4 (0.0-1.0)**  **Cont -0.2 (0.0-0.0) (P=0.003)**  **%Change in Body weight, median (95% CI) at 11 weeks** (27 week follow up data also available)  Int: 1.3 (0.6-3.2)  Cont: -0.6 (-1.6-0.6) (P=0.005) | **Change in total energy intake, MJ/d, median (95%CI)**  Int 0.7 (-0.3-1.2)  Cont -0.3 (-0.7-0.3) (P=0.08)  **Change in protein intake, g/d, median (95%CI)**  Int 5 (1-10)  Cont 2 (-5-4) (P=0.012) | **Mortality: 0-11 week, n (%)**  Int 8 (13)  Cont 4 (7)  **Change in ADL, mean score (95%CI)**  Int -0.1 (-1.3-1.07)  Cont -0.8 (-1.8-0.2) (P=0.26)  **Change in CPS, mean (95%CI)**  Int 0.2 (-1.1-0.5)  Cont 0.0 (-0.3-0.3) (P=0.25) |
| **Boffelli 2004 (29)**  **Italy**  **Setting**  Dementia unit  **Aim:**  To evaluate nutritional changes with 6 & 18 months of follow up after a nutritional intervention program. | **Participants:** Malnourished dementia unit patients  **N: 19** (40 screened for malnourishment, characteristics provided for 40)  **M/F:** 11/29  **Mean age (SD):** NR  **Nutritional status: 47.5% (19/40) malnourished albumin <3.5 g/L**  **Cognitive status mean MMSE score (SD)** 5.1 (5.9) **Dementia diagnosis:** diagnosed (no details)  **Dementia type:** 31 AD, 4 VD, 4 mixed & 1 Lewy bodies.  **Dementia stage:** Severe  **Acute illness:** NR | **Design: BA**  **Intervention: Type:** Nutritional program  **Provided by:** health worker (probably)  **Details:**  Modification of diet composition, quality & consistency of food based on preference and ability (swallowing, dental status)  Increased feeding time & assistance by nurses. Dining environment modification.  Nutritional supplements prescribed to those with low intake.  **Control: N/A**  **Duration of Int/ follow up:** 18 mo  **Compliance:** Not measured  **Individualised:** Yes  **Ethics obtained:** NR | **Body weight, kg, mean (SD)**  Baseline: 58.5 (11.7)  6 mo: 57.7 (11.2)  18 mo: 61.1 (12.5)  (P=0.5)  **BMI, mean (SD)**  Baseline: 22.7 (3.2)  6 mo: 22.8 (3.7)  18 mo: 23.7 (3.4)  (P=0.35)  **Serum albumin, g/dL, mean (SD)**  Baseline: 3 (0.3)  6 mo: 3.5 (0.3)  18 mo: 3.4 (0.3)  (P<0.05)  **No. malnourished (based on albumin)**  Baseline: 19/40  6 mo: 10/40 |  |  |
| **Carlsson 2009 (3)**  **Sweden**  **Setting**  Group-living facilities for People with dementia)  **Aim:**  To investigate the feasibility of serving drinkable yogurt enriched with probiotic bacteria to old people with dementia & also to test whether the drink could have any possible effect on constipation and body weight. | **Participants**: Dementia patients in 6 group-living facilities  **N:** 15 allocated (13 analysed)  **M/F:** 2/13  **Mean age (range)**:83.7 (69-93)  **Nutritional status:** BMI, mean (range) 26.2 (21-34)  Fluid intake, mean (SD) 1510 mL(318)  **Cognitive status:** NR  **Dementia diagnosis:** diagnosed (conventional clinical criteria)  **Dementia type:** 10 AD, 3 unspecified, 1 mixed & 1 hydrocephalus.  **Dementia stage:** NR  **Acute illness:** NR | **Design:** BA  **Int**: Type: Oral supplement (drinkable yogurt)  **Provided by:** Care home staff  Details: 1x200 ml drinkable yogurt (Verum Drickyogurt)/day (140 kcal, 6g protein, 1.6g fat, 26g CHO) served plus ordinary breakfast for 6 mo. Yogurt supplemented with lactobacillus rhamonsus LB21 & lactococcus lactis L1A.  **Compliance:** estimated to be 77% consumed.  **Cont**: N/A  **Individualised:** No  **Duration of Int/ follow up**: 6 months  **Ethics obtained:** Yes | **Change in body weight, kg, median (range)**  -3.9 (-9.2 – 3)  P<0.05 | **Energy intake (calculated from 7d dietary registration using computer software) kcal/d, mean (SD)**  Pre-Int 1454 (304)  6 mo. 1413(264) (no stats sig presented, there is also needs & deficit calculated)  P= 0.7(calculated)  **Fluid intake ml/d**  Pre-Int 1510 (318)  6 mo. 1549 (379)  P= 0.8 (calculated) | **Mortality**  2/15 died over 6 mo. |
| **Carlsson 2011 and Rosendahl 2006 (4,5)**  **FOPANU study**  **Sweden**  **Setting**  9 Residential care facilities  **Aim:**  To evaluate effects of high intensity functional exercise & timed protein/ energy drink on muscle mass | **Participants**: ADL dependent, cognitively impaired care homes residents  **N:** 191 randomised, 177 allocated (Exercise & Protein. 46, Exercise & placebo 45, Cont &Protein 50, Cont &placebo 50) (for protein 96 int vs 95 Cont)  **M/F:** 52/139  **Mean age (SD)**: 84.5 (6.4)  **Nutritional status BMI, mean (SD)** 24.9 (4.6)  **Cognitive status**  MMSE, mean (SD) 17.6 (5.1) **Dementia diagnosis:** NR 100/191 (52%) had dementia diagnosis but most were cognitively impaired based on MMSE  **Dementia type:** NR  **Dementia stage:** NR  **Acute illness:** NR | **Design: RCT (parallel, 4 arms)**  **Int**: Type: ONS (protein enriched drink plus/minus exercise)  **Provided by:** care staff & health workers  **Details:** milk-based protein-enriched drink (200 ml) (7.4g protein, 15.7g carbohydrate, 0.43g fat, = 408 kJ per 100g. plus High intensity functional exercise or sitting activity. Both activity types 45 min 5x2weeks for 13 weeks. Drink offered within 5 min of activity.  **Cont**: Placebo drink contained 0.2 g protein and 10.8 g carbohydrate, corresponding to 191 kJ per 100 g, offered within 5 min of activity. Plus exercise or sitting activity as in intervention.  **Compliance**: protein drink taken on 82% of occasions vs 78% for placebo  **Individualised:** No  **Duration of Int/ follow up**: 3 months  **Ethics obtained:** Yes | **Body weight, kg, mean (SD)** (groups combined here for Protein vs placebo)  **Baseline:**  Protein: 64.4 (12.1)  Placebo: 65.4 (15.2)  **3 months: (n=175)**  Protein: 64.7 (2.6)  Placebo: 65.5 (2.6)  **(Not significant)**  **6 months (n=163)**  Protein: 65.8 (3.4)  Placebo: 65.9 (3.2)  **P=not sig.**  **Intra cellular water (ICW), mean (SD)**  **Baseline:**  Protein: 12.8 (4.3)  Placebo: 12.5 (3.2)  **3 months:**  Protein: 12.4 (2.6)  Placebo: 12.6 (2.6)  **6 months:**  Protein: 12.4 (0.9)  Placebo: 12.4 (1.0) |  | **Mortality at 6 months**  **Protein: 7/96**  **Placebo: 5/95** |
| **Carver & Dobson 1995 (6)**  **UK**  **Setting**  **Psychiatric hospital/ elderly ward**  **Aim:**  To determine the effects of a dietary supplement on the body weight, triceps skinfold thickness & mid arm muscle circumference of low weight elderly demented hospital residents. | **Participants**: Underweight dementia patient in a psychiatric hospital ward  **N:** 46 randomised, 40 analysed (20 int., 20 Cont)  **M/F:** 10/36  **Mean age (SD)**: Int: F 80(10), M 69(9) Cont: F 79(10), M 68(7)  **Nutritional status: BMI** Int: F 18(1.3), M 17.4(1.7) Cont: F 18(1.4), M 17.5(1.8)  **Cognitive status: no details**  **Dementia diagnosis:** diagnosed (no details)  **Dementia type:** NR  **Dementia stage:** NR  **Acute illness:** NR | **Design: RCT (parallel)**  **Int**: Type: Dietary supplement  **Provided by: Nursing staff**  Details: 2 x 200ml oral supplement (Fortisip, Cow & Gate)/day. Providing 600 kcal/d.  **Cont**: 2 x 200 ml/d placebo drink providing 6 kcal/d plus the same vitamins as Fortisip, but no macronutrients.  Normal meals and assistance provided to both groups and no other changes.  **Compliance**: 19/20 of int consumed 100% vs 17/20 of placebo.  **Individualised:** No  **Duration of Int/ follow up**: 12 weeks  **Ethics obtained:** Yes | **Weight change, kg, mean (SD) at 12 weeks**  Int: 3.5 (1.8) compared to baseline  Cont: 0.6 (1.7)  P<0.001 (calculated)  **BMI, mean (SDs NR)**  Baseline: int. 17.9,  Cont 17.9.  12 weeks: int. 19.2, Cont 18.1.  **MAMC, c**m, mean (SD)  Int: Baseline 18.6 (1.6), 12 weeks 19.1(1.4) P<0.01  Cont: baseline 17.9(1.8), 12 weeks 17.9 (1.9)  **TSF** (mm, mean &SD)  Int: Baseline 7.3 (3.4), 12 weeks 8.8(3.9) P<00.001  Cont: baseline 7.8(1.8), 12 weeks 8.3 (3.5) |  |  |
| **De Sousa & Amaral 2012 (7)**  **Portugal**  **Setting**  **Geriatric unit of a psychiatric hospital**  **Aim:**  To evaluate the long term impact of a 3 week nutritional supplementation on the nutritional status of undernourished patients with probable AD | **Participants**: Malnourished mild dementia patients  **N:** 37 randomised(Int. 20, Cont 17), 35 analysed  **M/F:** 9/26  **Mean age (SD)**: int. 79.4 (6.9), Cont 78.4 (5.2)  **Nutritional status: BMI, mean (SD)** int.19.2 (2.7), Cont 19.5(1.3)  **Cognitive status: MMSE**, mean (SD) int.17 (7), Cont 18 (5)  **Dementia diagnosis:** DSM-IV & ICD criteria  **Dementia type:** AD  **Dementia stage:** Mild  **Acute illness:** No | **Design: RCT (parallel)**  **Int**: Type: ONS  **Provided by:** Health workers?  Details: 1 x 200 ml/day high protein energy-dense liquid NS drink (400 kcal, 42.8g CHO, 17.4 g fat & 18 g protein) plus standard dietetic advice. The drink was available in 2 flavours & consumed between breakfast & lunch.  **Cont**: Usual care + standard dietetic advice.  **Duration of Int/ follow up**: 3 weeks (3 months follow up)  **Compliance:** was Controlled by PI  **Individualised:** No  **Ethics obtained: Yes** | **Change in weight at 3weeks, kg, mean (SD)**  Int. +2.1(1.6), Cont 0.0(0.9) P<0.001  **Change in BMI at 3 weeks (mean, SD)**  Int. +0.9(0.7), Cont 0.0 (0.4) P<0.001  **Change in MNA (Mini Nutritional Assessment) at 3weeks, mean (SD)**  Int. 1.4 (0.8), Cont 0.0 (0.1) P<0.001 | **Changes in Barthel Index at 3weeks (measure level of independence out 100, higher score=more independence)**  Int. 0.0 (SD couldn’t be computed), Cont -0.7 (2.6) P not significant | **Mortality:**  Int 0 died, Cont 2 died  **Change in MMSE at 3weeks, mean (SD)**  Int. 0.0 (-), Cont 0.0 (-) |
| **Faxen-Irving 2002 (8)**  **Sweden**  **Setting**  Group-living for demented people  **Aim:**  To study the effects of nutritional intervention on body weight, cognition and ADL function in demented individuals. | **Participants**: people diagnosed with dementia living in two units  **N:** 36 (22 Int unit, 14 Cont unit) 33 analysed  **M/F:** 2/31  **Mean age (SD)**: int. 83 (4), Cont 85 (4)  **Nutritional status**  **Cognitive status: MMSE, mean (SD)** int.9 (6.6), Cont 8.5 (6.2)  **Dementia diagnosis: diagnosed (no details)**  **Dementia type:** AD (22%), vascular (20%), unspecified (58%)  Dementia stage: 12 mild, 10 moderate, 12 severe & 1 questionable.  **Acute illness:** NR | **Design: CCT (cluster)**  **Int**: Type: Nutritional & educational  **Provided by: care staff**  **Details**: * 2 x 200 ml ONS daily for 5 months; juicy supplement (170 kcal) in afternoon and balanced supplement (240 kcal) in eve.  * Education: staff attended 12 hr nutrition & diet program given by dietitians, physicians and external care personnel. Lectures were combined with practical exercises e.g. calculating BMI, thickening or enriching drinks.  **Cont**: usual care (no further details)  **Compliance:** 76% participants consumed all, rest consumed ~50%.  **Individualised:** No  **Duration of Int/ follow up**: 5 months  **Ethics obtained:** Yes | **Body weight (kg, mean & SD)**  Int: Baseline 55.4 (10.4), 6 months 58.8 (11.2)  Cont: baseline 62.2 (8.2), 6 mo 61.9 (10.4)  P=0.003 (comparing change between groups)  (9 mo after supplementation stopped, weight decreased by 2.6(3) kg in the remaining 15 Int unit residents) p<0.1  **BMI (mean, SD)**  Int: Baseline 22.2 (4.1), 6 months 23.5 (4.4)  Cont: baseline 24.6 (2.9), 6 mo 24.5 (3.8)  P=0.003 (comparing change) |  | **ADL (activities of daily living A-G, median, range)**  Int: baseline E (A-G), 6 months F (B-G)  Cont: baseline D (A-G), 6 months E (B-G)  **Clinical Dementia Rating, CDR total score (0-18)**  Int: Baseline 12.3 (5), 6 months 14.7 (3.6)  Cont: baseline 11.4 (4.6), 6 months 13.3 (3.6) |
| **Fiatarone Singh 2000 (9)**  **USA**  **Setting**  Nursing home (long term rehab centre)  **Aim:**  To test the hypothesis that the daily provision of a supplement with 20% additional energy would augment nutritional status as measured by total intake. (the study had 4 arms comparing exercise as well as nutrition to placebo) | **Participants**: Institutionalised elders  **N:** 50 (24 int., 26 Cont)  **M/F:** 19/31  **Mean age (SD)**: int. 85.6(5.9), Cont 89.2(4.1)  **Nutritional status: BMI, mean (SD)** int.25.4 (3.4), Cont 25.6 (0.5)  **Cognitive status: MMSE, mean (SD)** int.22.7(5.9), Cont 22.2 (5.1) - 26 (52%) had MMSE<24 **Dementia diagnosis:** NR  **Dementia type:** NR  **Dementia stage:** NR  **Acute illness: No** | **Design: RCT (parallel)**  **Int**: Type: Supplement  **Provided by: NR**  Details: 1x240 ml liquid supplement/day for 10 weeks offered in a choice of flavours (360 kcal, 60% CHO, 23% fat, 17% soy based protein & third of RDA vitamins & minerals). A soft nutritional bar was offered for those expressing distaste or gastric discomfort.  **Cont**: 1x 240 ml flavoured non-nutritive liquid.  **Duration of Int/ follow up**: 10 weeks  **Compliance:** 88% of supplement consumed. 1 participant consumed <10% & 79% consumed 80-100%  **Individualised:** No  **Ethics obtained:** Yes | **BMI, mean (SD)**  Int: Baseline 25.9 (3.4), 10 weeks 26.3 (3.8)  Cont: baseline 25.5 (3), 10weeks 25.3 (3)  p=0.024  **Body weight, kg, mean (SD)**  Int: Baseline 58.4 (11.5), 10 weeks 59.2 (11.5)  Cont: baseline 61.2 (10.5), 10 weeks 60.4 (11), p=0.021  **Total body water (using BIA), mean (SD)**  Int: Baseline 27.4 (4.3), 10 weeks 28.1 (3.8)  Cont: baseline 34.7 (14.5), 10 weeks 34.6 (15.5), p=0.298 | **Total energy intake (kcal/d, mean & SD)**  Int: Baseline 1562 (249), 10 weeks 1610(302)  Cont: baseline 1500 (315), 10 weeks 1430 (335)  **P=0.137**  **Water intake (g/d, mean &SD)**  Int: Baseline 1465 (259), 10weeks 1287 (317)  Cont: baseline 1355 (190), 10weeks 1335 (280) | **Mortality: 1/24 Int, 1/26 Cont**  **Katz index of ADL (higher scores indicate greater dependency)**  Int: Baseline 1.96 (1.6), 10weeks 2.27 (1.7)  Cont: baseline 1.72 (1), 10weeks 1.92 (1.1) |
| **Gregorio 2003 (10)**  **Spain**  **Setting**  **Nursing home**  **Aim:**  To ascertain the effects of an intervention with nutritional supplements on morbidity and mortality in a one year follow up. | **Participants**: Residents of 8 NHs with AD  **N:** 99 (Int 25, Cont 74)  **M/F:** 20/79  **Mean age (SD)**: int. 84.7 (3.8), Cont 85.6 (4.6)  **Nutritional status: BMI** 8.3% <19, 8.2% 19-21, 27.8% 21-23, 55.7% >23  **Cognitive status: MMSE, mean (SD)** 12.7 (5.3) **Dementia diagnosis:** NINCDS/ADRDA criteria  **Dementia type:** AD  **Dementia stage:** Moderate  **Acute illness:** Yes | **Design: RCT (parallel)**  **Int**: Type: supplement (assumed oral)  **Provided by: NR**  Details: A nutritional supplement (Nutrison) (125 kcal, 7.5 g protein, 14.5 g CHO, 4.2 fat). Not much details on timing or method of administration.  **Cont**: usual care  **Duration of Int/ follow up**: 12 months  **Compliance:** NR  **Individualised:** No  **Ethics obtained:** NR | **Change in BMI, mean**  After 1 yr Int: +1.6, Cont -0.3  **P=0.05**  **Change in MNA, mean**  After 1 yr Int: -0.2, Cont -3.2  **P=0.05** |  | **Mortality:**  4/25 death Int (16%), 17/74 deaths Cont (22%)  P=0.05  **Infections:**  Int: 47% had one infection.  Cont: 21% one infection, 30% two & 15% >3. |
| **Krikorian 2010a (11)**  **USA**  **Setting**  Community  **Aim:**  To assess effect of grape juice supplementation on memory in older adults with early age-related memory decline. | **Participants:** Older adults with mild cognitive impairment  **N:** 12 (5 int., 7 Cont)  **M/F: 8/4**  **Mean age (SD): 78.2(5)**  **Nutritional status: mean waist circum. Int.** 96.7, Cont 92.7.  **Cognitive status:** CDR 1.0 **Dementia diagnosis:** no dementia. MCI based on CDR  **Dementia type:** no dementia  **Dementia stage:** MCI  **Acute illness:** NR | **Design:** RCT (parallel)  **Int: Type:** Drink supplement  **Provided by:** Self?  **Details:** grape juice daily for 12 wks. Dosage 6-9 ml/kg body weight, in equal divided doses with 3 meals.  **Cont:** placebo drink formulated to match grape juice in colour, taste, total E (3kj/ml) & sugar profile but no juice or polyphenolic compounds.  **Duration of Int/ follow up:** 12 wks  **Compliance:** measured but NR.  **Individualised:** No  **Ethics obtained:** Yes | **Body weight** (kg, mean only)  Int: Baseline 79.4, 12 weeks 80.4  Cont: baseline 74.3, 12 weeks 74.9  **Waist circumference** (cm, mean only)  Int: Baseline 96.7), 12 weeks 97.5  Cont: baseline 92.7, 12 weeks 93.0 |  | **CVLT learning** (score, mean only, scores not explained)  Int: Baseline 35.2, 12 weeks 38.6  Cont: baseline 33.2, 12 weeks 33.2  ANOVA p=0.04  **S-PAL** (score, mean only)  Int: Baseline 2.8,  12 weeks 4.5  Cont: baseline 2.4, 12 weeks 2.0  P=0.12 |
| **Krikorian 2010b (12)**  **USA**  **Setting**  **Community**  **Aim:**  To investigate the effects of daily consumption of wild blueberry juice in older adults with early memory changes. | **Participants:** Older adults with mild cognitive impairment  **N:** 9 Int (7 Cont who were the same used in Krikorian 2010a)  **M/F:** NR  **Mean age (SD): int**. 76.2(5.2), Cont 80.2 (6)  **Nutritional status: mean waist circum.(SD)** 98 cm, 102(5) F & 92.5 (15)M.  **Cognitive status: CDR, mean (SD)** 0.88 (0.5) **Dementia diagnosis:** no dementia. MCI based on CDR  **Dementia type:** no dementia  **Dementia stage:** MCI  **Acute illness:** NR | **Design:** CCT  **Int: Type:** Drink supplement  **Provided by:** Self (probably)  **Details:** Commercially prepared wild blueberry juice in 1L amber glass bottles. Dose 6-9 ml/kg body weight/day. Given juice at baseline & week 6. Instructed to avoid berry fruits, juices & extract.  **Cont:** As for Krikorian 2010a (concord grape juice study)  **Duration of Int/ follow up:** 12 weeks  **Compliance:** measured but not reported  **Individualised:** No  **Ethics obtained:** yes | **Body weight (kg, mean & SD)**  Int: Baseline 80.3 (12), 12 weeks 80.3(?)  Cont: baseline 74.3, 12 weeks 74.9 (Cont data taken from concord grape study)  **Waist circumference (cm, mean only)**  Int: Baseline 98(11), 12 weeks 98.9  Cont: baseline 92.7, 12 weeks 93.0 (from concord grape study) |  | **CVLT data** not clear enough to use, exact subscales reported not clear.  **V-PAL,** data NR & contradictory. |
| **Krikorian 2012 (13)**  **USA**  **Setting**  Community dwelling older adults  **Aim:**  To assess the effects of grape juice supplement on cognition. | **Participants**: Older adults with MCI  **N:** 21 (10 int, 11 Cont)  **M/F:** 11/10  **Mean age (SD)**: 76.9 (6.1)  **Nutritional status:** NR  **Cognitive status:** MOCA **mean (SD)** Int 23.4 (2), Cont 22.8 (2). **Dementia diagnosis:** no dementia. MCI based on CDR  **Dementia type:** no dementia  **Dementia stage:** MCI  **Acute illness:** NR | **Design:** RCT (parallel)  **Int**: Type: Drink supplement  **Provided by:** Self?  Details: grape juice taken daily for 16 weeks divided between 3 meals. Dose 6.3-7.8 ml/kg body weight.  **Cont**: placebo drink matched grape juice in colour, taste, total E & sugar profile but no juice or polyphenolic compounds.  **Duration of Int/ follow up**: 16 wks  **Compliance:** measured but NR  **Individualised:** No  **Ethics obtained:** Yes | **Body weight (kg, mean & SD)**  Int: Baseline 76.7 (9), 16 weeks 76 (9)  Cont: baseline 74.9 (8), 16 weeks 74.1(8) |  |  |
| **Lauque 2000 (14)**  **France**  **Setting**  **Privately run nursing homes**  **Aim:**  To validate a nutritional intervention program for elderly people living in nursing homes. | **Participants:** Elderly residents of 8 nursing homes at risk of malnutrition (MNA 17-23.5)  **N:** 41 randomised, 35 analysed for groups C&B, outcomes provided here for combined groups (88 for full study which included 2 non-randomised groups not used here)  **M/F:** 5/30  **Mean age (SD): Int** 84.6 (5.5), Cont 84.7 (5.5)  **Nutritional status:** at risk of malnutrition (MNA 17-23.5)  **Cognitive status:** NR **Dementia diagnosis:** Int 85.7% (11/13) diagnosed with dementia, Cont 68.2% (15/22).  **Dementia type:** NR  **Dementia stage:** NR  **Acute illness:** No | **Design:** RCT (parallel)  **Int: Type:** Oral supplement  **Provided by:** NH staff  **Details:** nutritional supplements of 300-500 kcal in addition to regular meals. Four oral supplementation products offered in 3 different flavours (Clinutren, Nestle), soup, fruit or dessert each containing 120-200 kcal, 7.5-15 g protein and enriched with vitamins and minerals. Supplements given daily with meals  **Cont:** no supplements- regular meals  **Duration of Int/ follow up:** 60 days  **Compliance:** Average intake 393-430 kcal and compliance remained good.  **Individualised:** No  **Ethics obtained:** Yes | **BMI (mean, SD)**  Int: baseline 22.3(2.5), 60d 22.8 (2.5)  Cont: baseline 21.8 (4.2), 60d 21.3 (4.2)  **Weight (kg, mean, SD)**  Int: baseline 53.9(7.9), 60d 55.3 (9)  Cont: baseline 52.5 (11.3), 60d 51 (11.3) | **Total energy intake (kcal/d, mean & SD)**  Int: Baseline 1558 (216), 60d (including ONS) 1815 (393)  Cont: baseline 1583 (263), 60d 1562 (310)  P<0.001  **Protein intake**  **(g, mean, SD)**  Int: Baseline 57.4 (9.7), 60d 81.1 (18.4)  Cont: baseline 62 (13.6), 60d 62 (13.1)  P<0.001 | **Grip strength:**  **(kgW, mean, SD)**  Int: Baseline 4 (6.9), 60d 4.3(7.6)  Cont: baseline 5.9 (5.6), 60d 5.2(5.6)  Not significant |
| **Lauque 2004 (15)**  **France**  **Setting**  Geriatric wards & day centres  **Aim:**  To study the effects of ONS on body weight, body composition, nutritional status and cognition in elderly patients with AD | **Participants**: Geriatric ward and day centre residents at risk of malnutrition.  **N:** Int 46, Cont 45 randomised, Int 37, Cont 43 analysed  **M/F:** NR  **Mean age (SD)**: Int 79.5 (6), Cont 78.1 (4.8)  **Nutritional status: BMI, mean (SD) int** 22.2 (3.3), Cont 22.6(2.9) (MNA≤23.5)  **Cognitive status: MMSE, mean (SD) int.** 15.3(8.1), Cont 15.2 (8.5). **Dementia diagnosis:** NINCD/ADRDA criteria  **Dementia type:** AD  **Dementia stage:** Moderate  **Acute illness:** No | **Design:** RCT (parallel)  **Int**: Type: ONS  **Provided by:** Care workers  Details: A choice of 3 Clinutren products was offered ranging from 300-500 Kcal/day; soup (200 Kcal, 10 g protein/200 ml), dessert (150 Kcal, 12 g protein/ 150 ml), Clinutren 1.5 (300 Kcal, 11 g protein/200 ml). Products were savoury or sweet, liquid or creamy and warm or cold. ONS was discontinued at 3 months unless re-prescribed by GP.  **Cont**: usual care  **Duration of Int/ follow up**: 3 mo int (plus 3 months follow up)  **Compliance:** measured and supervised by dietician visits but results not reported  **Individualised:** No  **Ethics obtained: Yes** | **Weight Change (Kg, mean, SD) over 3 mo:** Int 1.9 kg (2.3), Cont 0.38 kg (2.3) **P=0.001**  **Over 6 mo:** Int 1.6 kg (3.4), Cont 0.67 kg (3.6)  **BMI Change (mean, SD) over 3 mo:** Int 0.8 (1.0), Cont 0.16 (0.9) **P=0.001**  **Over 6 mo:** Int 0.66 (1.4), Cont 0.29 (1.4)  **MNA Change (mean, SD) over 3 mo**: Int 3.4 (3.1), Cont 1.9 (3.5) **P=0.05**  **Over 6 mo:** Int 3.3(4.4), Cont 2.5 (4.2) | **Energy intake change (Kcal/d, mean, SD) over 3 mo:** int., 291 Kcal (418),  Cont -1 (466) **P=0.001**  **Over 6 mo:** Int 87 Kcal (419), Cont 178 Kcal (503) | **MMSE Change (mean, SD) over 3 mo:** Int 0.33 (2.9), Cont -0.41 (2.6)  **Over 6 mo**: Int -1.36 (2.9), Cont -1.8 (3.6)  **ADL Change (mean, SD) over 3 mo:** Int -0.2 (1.1), Cont -0.28 (0.9**)**  **Over 6 mo:** Int -0.5 (1.2), Cont -0.6 (1.2)  **EBS Change over 3 mo:** Int --0.44 (1.7),  Cont -0.37 (5.1)  **Over 6 mo** Int -0.97 (3.2),  Cont -0.63 (5.3) |
| **Manders 2009 (16)**  **The Netherlands**  **Setting**  **Nursing homes**  **Aim:**  To determine whether a nutrient-dense drink has a positive effect on mental and physical function of institutionalised elderly people. | **Participants**: Institutionalised elderly persons.  **N:** 176 allocated, 111 analysed (78 int., 33 Cont)  **M/F:** 54/122  **Mean age**: 83  **Nutritional status: BMI, mean (SD)** Int. 25.3 (3.6) Cont 25 (3.5)  **Cognitive status: mean MMSE (range)** Int. 22(12-27), Cont 21 (12-26) **Dementia diagnosis:** NR  **Dementia type:** NR  **Dementia stage:** NR but MMSE had to be ≥10  **Acute illness: No** | **Design: RCT (parallel)**  **Int**: Type: Supplement  **Provided by: NR**  **Details:** 2 x 125ml/d nutrient dense drink (250kcal/d, 8.75g/d protein, providing 25-175% US RDA of micronutrients). Two flavours offered.  **Cont**: placebo drink with water, sweetener, cloudifier, thickener, flavouring, colour, sweetener. Offered in same 2 flavours.  **Duration of Int/ follow up**: 24 weeks  **Compliance:** 67% median compliance in 111/176 completing the study  **Individualised:** No  **Ethics obtained:** Yes | **Change in weight** at 24 weeks: Kg, mean (SD)  Int: 0.8 (3.6)  Cont: -0.8 (3.3)  P=0.04  **Change in calf circumference** at 24 weeks: cm, mean (SD)  Int: 0.3 (2.1)  Cont: -0.6 (2.4)  P=0.051 |  | **Mortality:**  6/176 deaths. NR by study arm.  **BI Change** at 24 wks, median (p10-p90) Int. 0.0 (-4.0-3.0), Cont 0.0 (-4.0-3.0) P=0.75  **Grip strength change,** kgf, mean (SD) Int: -0.5 (3.5),  Cont: -1.5 (3.8)  P=0.23  **ADAS-cog change** median (p10-p90)  Int: 0.0 (-5-8)  Cont: 1 (-5.6-6)  p=0.85 |
| **Navratilova 2007 (17)**  **Czech Republic**  **Setting**  **Institutions?**  **Aim:**  To assess the relationship between nutritional support and the improvement or the maintenance of patients’ health. | **Participants**: AD patients from 7 Czech institutions  **N:** 100 (50 int, 50 Cont)  **M/F:** NR  **Mean age**: NR  **Nutritional status:** NR  **Cognitive status:** NR  **Dementia diagnosis:** ICD-10 criteria  **Dementia type:** AD  **Dementia stage:** NR  **Acute illness:** NR | **Design: RCT (parallel)**  **Int**: Type: Oral supplement  **Provided by:** NR  **Details:** After a year of standard voluntary food intake, Int group received usual food plus daily nutritional supplement (Nutridrink, 600 Kcal/d, 24g protein/d, 74g CHO/d, 23 g/day fat.  **Cont**: ordinary food, no supplement.  **Duration of Int/ follow up**: 1 year  **Compliance:** NR  **Individualised:** No  **Ethics obtained:** NR | No numbers reported just stated that “No statistically significant change in BMI or weight” | **Change in Energy intake, Kcal:** +51 Kcal (excluding supplements). No separate group figures provided or P values reported. Just stated “Statistically significant  increase (+51Kcal) in  overall daily energy  intake in OLNS group”  **Change in Protein intake, g:** +8 g  **Change in CHO intake, g:** +27 g | **Change in MMSE at 1 year, score, mean (SD)**  Int -1.3 (0.8), Cont -3.7 (0.6) P=0.024 |
| **Pivi 2011 (18)**  **Brazil**  **Setting**  **NR**  **Aim:**  To evaluate if there is a difference between nutrition education and oral nutritional supplementation on nutritional status in patients with AD. | **Participants**: Elderly patients with probable AD  **N:** supplement 26, Cont 27  **M/F:** 25/53  **Mean age**: 75.2  **Nutritional status:** NR  **Cognitive status: MMSE, mean** 12.8 (Edu), 11.6 (suppl), 12.6 (Cont **Dementia diagnosis:** DSM-IV and CDR  **Dementia type:** AD  **Dementia stage:** 23 mild, 31 mod, 24 severe (based on CDR)  **Acute illness:** NR | **Design: RCT (parallel, 3 arms, only supplement arm reported here)**  **Int**: Type: Supplementation  **Provided by:** NR  Details: Int ONS twice daily (Ensure with FOS®, Abbott Nutrition, providing 680kcal/d, 25.6g/d protein) for 6 mo in addition to usual diet  **Cont**: usual care plus monthly nutritional assessment.  **Duration of Int/ follow up**: 6 mo  **Compliance:** NR  **Individualised:** No  **Ethics obtained:** Yes | **BMI, change after 6 mo: mean**  SG: +6.6, Cont: -2.2  P<0.001  **Weight change, mean Kg**  SG: 6.7, Cont: -2.2  P<0.001  **Arm circumference, cm**  SG: 5.44, Cont: -0.4  P=0.002  **TSF, mm**  SG: 1.4, Cont: 2.2  P=0.140 |  |  |
| **Planas 2004 (19)**  **Spain**  **Setting**  **Dementia care day centre**  **Aim:**  To evaluate if a nutritional supplementation with or without micronutrient enhancement prevent weight loss and the progression of the disease in mild AD patients. | **Participants**: Mild AD patients in an Alzheimer day centre  **N:** 44 (23 int., 21 Cont)  **M/F:** 20/24  **Mean age (SD)**: 72.5 (10.7) int., 76.7 (5.5) Cont.  **Nutritional status: BMI, mean (SD)** int. 25.4 (4.4), Cont 24.4 (2.6).  **Cognitive status: GDS, mean (SD)** int. 4.7 (1.1), Cont 4.7 (1.0)  **Dementia diagnosis:** NINCDS-ADRDA criteria  **Dementia type:** AD  **Dementia stage:** Mild  **Acute illness:** NR | **Design:** RCT (parallel, treated as BA for this review since both groups had supplements)  **Int**: Type: Nutritional supplement with micronutrients  **Provided by:** care staff  **Details:** Participants ate 3 regular spaced meals/day plus twice/ day a 250 energy dense and protein rich liquid supplement (500 Kcal/day, 45% CHO, 25% fat & 30% protein) with added micronutrients administered between meals.  **Cont**: same supplement but with no added micronutrients  **Duration of Int/ follow up**: 6 months  **Compliance:** estimated to be ~90%  **Individualised:** No  **Ethics obtained:** Yes | **BMI, mean (SD)**  Int: Baseline 25.4 (4.4),  6 mo 26.5 (4.5).  Cont: Baseline 24.4 (2.6),  6 mo 26.0 (3.5)  P=0.59 (between gp diff)  P=0.055 (within gp diff)  **TSF, mm, mean (SD)**  Int: Baseline 17.7 (5.9),  6 mo 18.5 (6.0).  Cont: Baseline 16.8 (3.4),  6 mo 18.1 (3.4)  P=0.88 (between gp diff)  P=0.055 (within gp diff)  **MAC, cm, mean (SD)**  Int: Baseline 28.7 (3.1),  6 mo 29.1 (3.1).  Cont: Baseline 28.9 (2.9),  6 mo 29.5 (3.0)  P=0.64 (between gp diff)  P<0.001 (within gp diff) | **Daily energy intake, Kcal/d, mean (SD)**  Int:  Baseline 1590 (210),  6 month 1990 (180).  Cont:  Baseline 1600 (180),  6 month 2000 (230)  P (between group diff)= 0.39  P (within group diff) <0.001 | **MMSE, score, mean (SD)**  Int:  Baseline 19.1 (10.2),  6 month 18.2 (10.7).  Cont:  Baseline 18.2 (10.1),  6 month 15.1 (10.9)  P (between group diff)= 0.99  P (within group diff)= 0.96 |
| **Scheltens 2010 & 2012 (20,21)**  **Belgium,**  **Germany,**  **Netherlands, UK, USA**  **Setting**  AD Treatment Centres  **Aim:**  To determine the effect of a medical food on cognitive function in people with mild AD. | **Participants**: People with mild AD, attending AD treatment centres  **N:** 225 (112 Int)  **M/F:** Int.54 (51% male), Cont: 52 (42% male)  **Mean age (SD)**:Int. 74.1 (7.2), Cont: 73.3 (7.8)  **Nutritional status:** BMI, kg/m^2^**, mean (SD)** Int (n=106) 26.2 (4.8), Cont (n=106) 26.2 (3.5)  **Cognitive status:** MMSE, **mean (SD)** int.(n=105): 23.8 (2.7), Cont (n=105): 24.0 (2.5)  **Dementia diagnosis:** NINCDS-ADRDA Criteria  **Dementia type:** AD  **Dementia stage:** mild  **Acute illness:** NR | **Design: RCT** (ITT analysis)  **Int**: Type: Medical Food Supplement  **Provided by:** Self  **Details:** Int: 1x125ml/d tetrapack (‘Souvenaid’), choice of 2 flavours.  Cont: 1x125ml/d tetrapack, choice of 2 flavours, in identical packaging, isocaloric, isonitrogenic, similar in flavour & taste to Souvenaid  **Duration of Int/ follow up**: 12 wks & 12 wk extension  **Compliance:** Participants recorded daily consumption (0, ¼, ½, ¾). Non-compliance was none taken on >25% days or average intake <70% prescribed. Int: 96% compliant, Cont: 95% compliant.  **Individualised:** No  **Ethics obtained:** yes | **BMI, kg/m^2^, marginal mean, 12 weeks**  Int (n=98): 26.3 (95%CI: 25.5, 27.1)  Cont (n=97): 26.2 (95%CI: 25.4, 27.0). *p=*0.39  **BMI, kg/m^2^, marginal mean, 24 weeks**  Int (n=76): 26.4 (95%CI: 25.6, 27.2)  Cont (n=76): 26.2 (95%CI: 25.4, 27.0). *p=0.07*  **QoL-AD, mean score, 12 weeks**  Int (n=101): 34.8 (SD: 4.2)  Cont (n=99): 35.6 (SD: 4.3). *p=0.305* |  | **MMSE, mean, 12 wks. I**nt (n=99): 24.1 (SD: 3.5)  Cont (n=96): 24.0 (SD: 3.4). *p=0.53*  **ADCS-ADL, mean, 12 wks** Int (n=101): 62.3 (SD: 10.7)  Cont (n=99): 62.6 (SD: 11.4). *p=0.31*  **WMS-r delayed verbal recall, mean, 12 wks**  Int (n=100): 40% improved, 19% deteriorated, 41% no change  Cont (n=98): 24% improved, 34% deteriorated, 42% no change. *p=0.02*  **WMS-r, immediate verbal recall, mean, 12 wks**  Int (n=100): 50% improved, 31% deteriorated, 19% no change  Cont (n=98): 40% improved, 45% deteriorated, 15% no change. p*=0.13*  **24 weeks:** *p=0.046 (scores NR)*  **ADAS-cog, mean, 12 wks** Int (n=101): 25.9 (SD: 7.7)  Cont (n=99): 25.8 (SD: 7.8). *p=0.83* |
| **Simmons 2010 (22)**  **USA**  **Setting: Long-term care facilities.**  **Aim:**  To determine the cost-effectiveness of supplements relative to offering residents’ snack foods and fluids between meals to increase caloric intake. | **Participants**: care home residents receiving nutritional supplements  **N:** 63 (Int 1 18, Int 2 25, Cont 20)  **M/F:** 24/39  **Mean age, sd**: 86.9 (11.3)  **Nutritional status: BMI <20 kg/m^2^, n(%):** 15 (24)  **E intake, kcal, mean (SD):**  Int 1. mealtime: 990 (376)  Betw meals: 206 (206)  Total: 1196 (427)  Int 2. Mealtime: 1080 (405)  Betw meals: 113 (134)  Total: 1193 (419)  Cont: mealtime: 1019 (423)  Betw meals: 130 (129)  Total: 1149 (461)  **Cognitive status, mean MMSE:** 14.1 (8.9) **Dementia diagnosis:** MMSE assessed by researchers  **Dementia type:** NR  **Dementia stage:** NR  **Acute illness:** NR | **Design:** RCT (parallel)  **Int**: Type: supplementation  **Provided by:** research staff  **Details:** Int 1: supplements provided twice daily, between meals, consistent with food order of physician/dietitian,  Int 2: Snacks provided twice a day between meals, consistent with dietary specifications in medical notes  **Both Int groups:** assistance provided to enhance intake and independence according to standardised protocols  **Cont**: usual care (not described)  **Duration of Int/ follow up**: 6 weeks at 5 d/wk  **Compliance:** research staff noted amounts offered and consumed, and assistance required but NR  **Individualised:** partly, choice of flavours (supplements) and foods offered (snacks)  **Ethics obtained:** yes | **Weight, lbs, mean change baseline/6 weeks:**  **Int 1 (supplements):** 2.01 (SD: 5.15)  **Int 2 (snacks):** 0.04 (2.75)  **Cont:** 0.53 (4.32)  Cont v Int 1, *p=*0.34  Cont v Int 2, *p=*0.66  *p values calculated by reviewers* | **Caloric intake, kcal, mean (SD) and mean change from baseline:**  1. Supplement group:  mealtime: 805 (326); -124  Between meals: 391 (339); +151  Total: 1196 (351); +28  2. Snack group:  mealtime: 975 (407); -96  Between meals: 304 (245); +163  Total: 1279 (524); +67  3. Cont group:  mealtime: 1025 (371); +5  Between meals: 73 (93); -70  Total: 1098 (415): -65  ***Mealtime***  Cont v Int 1, *p=*0.05  Cont v Int 2, *p=*0.67  ***Between-meals***  Cont v Int 1, *p=*0.0001  Cont v Int 2, *p<0.0001*  ***Total***  Cont v Int 1, *p=*0.43  Cont v Int 2, *p=*0.20  *p values calculated by reviewers* | **Staff time,** **between meals,** *min/resident/offer*  Int 1: 13.8 (SD: 12.5)  Int 2: 12.3 (SD: 10.0)  Cont: N/R (but Ints required increase of 1.7 (SD: 3.2) mins/resident/offer over usual care)  **Staff time,** **meal-times,** remained stable across all three groups at <10 mins/resident/meal  **Mean change in costs from baseline to post-int for between meal interventions (Includes product cost and staff time), $/day:**  Int 1: -0.03  Int 2: +2.10  Cont: +2.06  Cont v Int 1, *p<0.001*  Cont v Int 2, *p<0.001* |
| **Stange 2013 (23)**  **Setting: Nursing home**  **Aim**  To investigate the effects of a low-volume energy and nutrient-dense ONS on nutritional status, functionality and quality of life of nursing home residents. | **Participants**: Residents of 6 nursing homes at risk of malnutrition.  **N:** 87 (45 Int, 42 Cont)  **M/F:** int.5/37; Cont 2/33  **Mean age (SD)**: int.87 (6); Cont 86 (7)  **Nutritional status:** BMI, kg/m^2^, **mean (SD)** Int.23 (3.4), Cont 22.5 (3.1)  **Cognitive status, MMSE, mean (range)**  Int. 6 (1-14), Cont 5 (0-18) **Dementia diagnosis:** NR (diagnosis obtained from case notes). Int.76.2%; Cont 65.7%  **Dementia type: not stated**  **Dementia stage:** mild to severe cognitive impairment  **Acute illness: NR** | **Design: RCT**  **Int**: Type: ONS  **Provided by: care home staff**  Details: 2x125ml bottles of ONS/resident/day, given between meals. Choice of flavours, staff encouraged drinking.  **Cont**: Usual care which included snacks and/or provision of ONS if prescribed by physician or family.  **Duration of Int/ follow up**: 12 weeks  **Compliance:** assessed by nursing staff who documented amounts consumed as a proportion (0, ¼, ½, ¾, all). Further checks by research staff conducted 3-7x/week.  **Individualised:** no, apart from choice of flavours  **Ethics obtained: Yes** | These outcomes Int n=42, Cont n=35  **Weight change, kg, mean (SD):**  *Int:* +1.2 (2.4),  Cont: -0.5 (2.3) *p*=0.002  **BMI, kg/m^2^, mean (SD):**  *Int:* 23.5 (3.3)  *Cont:* 22.3 (3.1); *p*=0.002  **MNA-SF, mean (range):**  *Int:* 10 (8-11)  *Cont:* 9 (8-10); *p*=0.8  **UAC, cm, mean (range):**  *Int:* 25.0 (22.4-27.0)  *Cont:* 24.8 (22.8-27.0); *p*=0.015  **Calf circumference, cm, mean (SD):**  *Int:* 31.0 (4.4)  *Cont:* 30.3 (3.3); *p*=0.018 | (Energy & protein intake: n=35 Int; n=31 Cont)  **Energy intake, kcal/day, mean (SD):**  *Int:* 1615 (442)  *Cont:* 1496 (299); *p*=0.211  **Protein intake, g/day, mean (SD):**  *Int:* 54.9 (18.2)  *Cont:* 48.0 (12.1); *p*=0.077 | **Mortality**: 10 deaths following start of study, no further deaths during the study  **MMSE, mean (range):**  *Int (n=41):* 7 (0-14)  *Cont (n=35):* 6 (0-20); *p*=0.430  **ADL, mean (range):**  *Int (n=42):* 25 (5-40)  *Cont (n=35):* 25 (5-65); *p*=0.979 |
| **Wouters-Wesseling 2002 (24)**  **Netherlands**  **Setting**  Nursing homes  **Aim:**  To investigate the feasibility of  Supplementation with a complete nutrient-enriched low volume liquid nutrition supplement especially intended for use in the elderly. | **Participants**: Nursing home residents with dementia and normal BMI  **N:** Int 21, Cont 21 allocated, Int 19, Cont 16 analysed  **M/F:** 4/31  **Mean age (SD)**: Int 85.3 (8.4), Cont 78.7 (8.8)  **Nutritional status: BMI, mean (SD)** int. 20.7 (3.2), Cont 20.7 (2.7).  **Cognitive status:** NR **Dementia diagnosis:** diagnosed (no details)  **Dementia type:** 17 AD, 5 multi-infarct, 13 not specified.  **Dementia stage:** NR  **Acute illness:** No | **Design: RCT (parallel)**  **Int**: Type: ONS  **Provided by: Nursing staff**  Details: 2 X 125 ml tetra packs/ day of liquid nutrition supplement (273 Kcal, 8.5 g protein, 39.6 CHO, 8.9 g fat) between main meals in two different flavours (orange=peach and blackberry) for 3 months.  **Cont**: same amount and flavour of a placebo drink.  **Duration of Int/ follow up**: 12 weeks  **Compliance:** 228 ml (SD 20.5) 91%/ day (not clear if for both groups or Int only)  **Individualised:** No  **Ethics obtained:** Yes | **BMI, mean (SD)**  Int: Baseline 20.7 (3.2),  3 mo 21.2 (2.9).  Cont: Baseline 20.6 (2.7), 3 mo 20.4 (3.0)  **Weight, Kg, mean (SD)**  Int: Baseline 51.7 (10.2),  3 mo 53.1 (10.1).  Change +1.4 (2.4)  Cont: Baseline 53.4 (8.8),  3 mo 52.6 (8.3)  Change -0.8 (3.0)  p=0.02 |  | **Mortality:**  **Int 1/21**  **Cont 2/21**  **Diarrhoea** (number of days with diarrhoea)  **Median (range)**  Int:  2 (0-18)  Cont:  1 (0-21)  **BI median (range)**  Int:  Baseline 4 (0-20),  3 month 4 (0-20).  Cont:  Baseline 5.5 (1-15),  3 month 5.0 (1-15) |
| **Wouters-Wesseling 2006 (25)**  **Netherlands**  **Setting**  Nursing homes  **Aim:**  To investigate whether liquid nutrition supplement use early after onset of acute infection can prevent weight loss in elderly nursing home residents. | **Participants**: Residents of psychogeriatric nursing homes with acute infection.  **N:** Int 19, Cont 20 allocated, int 18, Cont 16 analysed  **M/F:** 5/29  **Mean age (SD)**: Int 83.8 (6.9), Cont 81.6 (7.5)  **Nutritional status: BMI, mean (SD)** int. 24.4 (3.6), Cont 24.8 (4.9)  **Cognitive status:** NR **Dementia diagnosis:** NR (residents of psychogeriatric nursing homes)  **Dementia type:** NR  **Dementia stage:** NR  **Acute illness:** Yes | **Design: RCT (parallel)**  **Int**: Type: ONS  **Provided by: Care home staff**  Details: 200 ml/day ONS (309 Kcal, 38g CHO, 13g fat, 11g protein) for 5 wks starting at antibiotic prescription  **Cont**: usual care (dietary int started if weight loss was observed)  **Duration of Int/ follow up**: 5 wks  **Compliance:** measured but NR  **Individualised:** No  **Ethics obtained:** Yes | **Weight change, Kg, mean (SD)**  Int: 0.78 (1.7),  Cont: -0.38 (2.0)  P=0.04  **TSF Change, mm, mean (SD)**  Int: -0.45 (1.3)  Cont: -0.38 (1.6)  P=0.88  **AMC Change, cm, mean (SD):**  Int: -0.37 (1.1)  Cont: 0.26 (1.2)  P=0.13 | **Change in Energy intake, Kcal, mean (se)**  **Int:**  -109 (1567)  **Cont:** 5.6 (269)  (no baseline assessment but week 1 & week 5 based on 3 days main meal and the rest estimates) | **Change in ZIG independency score** (higher scores= greater dependency) **mean (SD)**  **Int:**  1.56 (3.4)  **Cont:**  1.56 (3.2) |
| **Young 2004 & 2005 (26,27)**  **Canada**  **Setting**  Nursing home  **Aim:**  To examine whether providing a midmorning  nutrition supplement increases habitual energy intake in  seniors with probable Alzheimer’s disease (AD) and to investigate  the effects of body weight status and cognitive and  behavioural function on the response to the Int | **Participants:** Residents of 7 AD units  **N:** Int 1 15, Int 2 19  **M/F:** 7/27  **Mean age (SD):** Int 1, 87.6(4.9), Int 2 88.4 (3.5)  **Nutritional status: BMI, mean (SD)** Int 1, 23.4 (3.9), Int 2, 24.1 (3.7)  **Cognitive status:** GDS_1_, mean (SD) 5 (1.1) **Dementia diagnosis:** by qualified clinician  **Dementia type:** AD  **Dementia stage:** NR  **Acute illness:** No | **Design: RCT** (crossover**,** treated as parallel RCT for phase 2 for primary outcomes)  **Int: Type**: Int 1.Supplement,  Int 2. Food service  **Provided by:** care home staff  **Details: Int 1, Supplement: 21 d of** daily nutritional supplement (3/4 nutrition supplement bar and fruit juice (apple, orange, cranberry, or prune), providing 258 kcal, protein 17%, fat and CHO 61%) between breakfast and lunch.  **Int 2, high-carbohydrate dinner:** replacing 12 ‘‘traditional’’ dinners on alternate days with high CHO meals comparable to traditional dinners in protein. Int foods included usual breakfast foods such as juice, bread, jam, cereal, cheese.  **Cont:** Habitual intake (21 days measurement at baseline)  **Duration of Int/ follow up:** 21 days (whole study 12 weeks)  **Compliance:** 78.8% (SD 22%) supplement intake.  **Individualised: No**  **Ethics obtained: Yes** | **Change in weight** (mid-morning supplement vs baseline measurement) n=13 (phase 2 only, not crossover)  +1.38 Kg (1.0) P=0.0003  **Weight change for HCD Int is not presented by phase** | **Change in Energy intake** (mid-morning supplement vs baseline measurement) n=34 (crossover)  +136.1 Kcal/day (187)  P<0.001  **Change in Protein intake** (mid-morning supplement vs baseline measurement) n=34 (crossover)  +5.8 g/day (7.3)  P<0.001  **Change in CHO intake** (mid-morning supplement vs habitual intake) n=34 (crossover)  +23.9 g/day (29.7)  P<0.001  **Change in Energy intake** (HCD vs baseline measurement) n=32 (crossover)  +109.6 Kcal/day (141.8)  P<0.001 |  |
| **Food modification/ swallowing problems intervention studies** | | | | | |
| **Bautmans 2008 (30)**  **Belgium**  **Setting:**  Nursing home  **Aim:**  To investigate the feasibility and effect on swallowing of cervical spine mobilization in frail elderly dysphagic nursing home residents with severe dementia | **Participants**: NH residents with dementia and dysphagia  **N:** Int 8, Cont 7 analysed  **M/F:** 6/9  **Median: (by gender)** Int F 91, M 82**,** Cont F85, M 83  **Nutritional status: BMI (median)**  Int F 21.4**,** M 27, Cont F24.2, M 21.8  **Cognitive status:** MMSE (median) Int F 7, M 12, Cont F 10.5, M 7 **Dementia diagnosis:** diagnosed but no details  **Dementia type:** AD  **Dementia stage:** Severe  **Acute illness:** No | **Design: RCT (crossover)**  **Int**: Type: Cervical spine mobilization for dysphagia  **Provided by: physiotherapist**  **Details**: Int: gentle cervical spine mobilization to correct posture, by therapists familiar with the residents (3 sessions/wk each lasting 20 min)  **Cont**: Socializing visit by therapists  **Duration of Int/ follow up**: 1 week  **Complianc**e**:** 90% of sessions were conducted  **Individualised: No**  **Ethics obtained: Yes** |  | **Dysphagia limit: (maximal bolus of water that can be swallowed in a single movement), median (25%-75%)**  Baseline: 3 ml (1-10)  After 1 session: 5 ml (3-15)  P=0.01 compared to Cont  1 week: 10 ml (5-20) P=0.03  Cont data is not presented in numbers but just in figure |  |
| **Beck 2010 (28)** | **As above** |  |  |  |  |
| **Boffelli 2004 (29)** | **As above** |  |  |  |  |
| **Germain 2006 (31)**  **Canada**  **Setting**  **Long term care facility**  **Aim:**  To evaluate the nutrient intake of frail institutionalised elderly persons with dysphagia & to assess the impact of a nutritional care program on dietary intake and weight | **Participants**: elderly residents with known dysphagia, weight loss & cognitive decline  **N:** 17 (8 int., 9 Cont)  **M/F:** 7/10  **Mean age (SD)**: int. 82.5 (4.4), Cont 84.6 (3.8).  **Nutritional status: BMI, mean (SD) int.** 22.4 (3.9), Cont 21.2 (2.3)**.**  **Cognitive status: NR** **Dementia diagnosis: (14/17) diagnosis from patient’s notes**  **Dementia type:**8 AD, 6 other dementia  **Dementia stage: NR**  **Acute illness: NR** | **Design: RCT (parallel)**  **Int**: Type: modified diet  **Provided by:** care home staff  **Details**: Reformed foods (pureed fruits, vegetables, and desserts along with pureed and minced meats), thickened beverages, as needed, and dietary supplements when necessary. Individualised based on preference.  **Cont**: traditional modified textured diets presenting 3 levels: Minced-70, Minced-3, and Pureed diet and honey-thickened beverages (not systematically controlled).  **Duration of Int/ follow up**: 12 wks  **Compliance**: NR  **Individualised:** Yes  **Ethics obtained:** Yes | **Weight change, kg, mean (SD)** at 12 weeks Int: +3.9 (2.3),  Cont -0.79 (4.18)  **P=0.02** | **E intake change at 12 weeks (kcal/d, mean & SD)**  Int: +611(408)  Cont: 81 (169)  **P=0.03** |  |
| **Jean 1997 (33)**  **USA**  **Setting**  Nursing home  **Aim:**  To implement a finger food menu with selected residents to determine whether this could improve their overall nutritional status and independent feeding skills. | **Participants**: cognitively impaired NH residents with limited use of eating utensils and poor intake  **N:** 12  **M/F:** NR  **Mean age(SD)**: NR  **Nutritional status:** had weight loss (no details)  **Cognitive status:** cognitively impaired (no data) **Dementia diagnosis:** NR  **Dementia type**: half had AD & half other dementias.  **Dementia stage:** NR  **Acute illness:** NR | **Design: BA**  **Int**: Type: Finger food menu  **Provided by: health worker**  Details: four-week cycle menu. Training for nursing and dietary staff on the rationale for the finger food diet including fundamental concepts of the menu alterations and actual presentation of the meals  **Cont**: N/A  **Duration of Int/ follow up**: 6 months  **Compliance:** NR  **Individualised:** No  **Ethics obtained:** no | **Weight loss arrest (number of participants with weight loss arrest after 6 months)**  10/12 participants  **Discontinuation of supplements:**  In 25% of cases it was found that high E and protein supplements could be discontinued. |  | **Feeding skills independence: (a scale to measure feeding assistance)**  All of the residents became more independent with feeding skills. 3 patients who initially required feeding assistance were able to feed themselves entirely. |
| **Keller 2003 (36)**  **Canada**  **Setting**  4 Special care units in 2 long term care facilities  **Aim:**  To determine whether body weight can be maintained or improved in dementia residents of special care units using a comprehensive intervention strategy. | **Participants**: patients with dementia and orally fed from 4 special care units  **N:** 82 (33 int., 49 Cont)  **M/F:** 31/51  **Mean age (SD)**: 79.7(7.2) int., 79.8(7.4) Cont  **Nutritional status: BMI, mean:** int. 24.9 M & 24.8 F, Cont 23.9 M& 25 F  **Cognitive status: MMSE, mean (SD)** int.9(5.9), Cont 8.3(6.6)  **Dementia diagnosis:** diagnosed (dementia unit)  **Dementia type:** AD (84.8% int. & 69.4% Cont) and others  **Dementia stage:** NR  **Acute illness:** NR | **Design: CCT**  **Int** Type: food service & staffing (comprehensive Int)  **Provided by:** Care home staff & dietitian  **Details:** Enhanced dietitian time and enhanced menu:  * 1^st^ 9 months = Cont, standard care  * 2^nd^ 9 months: dietetic time enhanced as needed, menu modified (snack rotation included portable high-energy snacks, super-mashed potato as a standard, high protein milk with meals, blended breakfast for those needing breakfast supplement). Also increased staff awareness & nutrition communication  * following 12 months: enhanced menu continued, dietitian as baseline  **Comparison sites**: standard nutritional care menu; 3 meals and 2 snacks. Dietetic time 15min/resident/month.  **Duration of Int/ follow up**: 30 mo  **Compliance:** NR  **Individualised:** yes  **Ethics obtained:** Yes | **Weight change over whole 30 months period, %, mean (SD)**  Int +4.8% (0.7%),  Cont -4.5% (0.9%)  P<0.001  Int: 27.3% gained >5% body weight & 6.1% lost>5%  Cont 6.8% gained >5% & 36.4% lost>5% |  | **Mortality: %**  Int: 6.1%  Cont: 16.3%  **Infections (mean, SD):**  Int 2.3(1.6)  Cont 2.6 (1.7)  **Dietitian time, mins, mean (SD)** (for the 9 months int period)  Int 533.3 min (94.5)  Cont 17.6 (21.6)  P<0.001  **Hospital days (mean, SD)** (for the 9 month int period)  Int 0.85 (2.9)  Cont 0.4 (1.3) |
| **Kenkman 2010 (37)**  **UK**  **Setting**  Care homes  **Aim:**  To assess the health, wellbeing & nutritional status of a population of older people living in UK residential care, and to assess the feasibility of measuring the effects of a change in provision of food and drink in this context. | **Participants:** Residents of 6 care homes in Norfolk  **N:** 105 allocated (Int 57 from 3 NH, Cont 48 from 3NH) 63 analysed.  **M/F:** 31/74  **Mean age (SD):** 86 (6.7) int, 88 (6.8) control  **Nutritional status: BMI, mean (SD)** Int. 25.8 (5.3), Cont 24.7 (4.5)  **Cognitive status: MMSE, mean (SD)** int19 (5.6), Cont 17(6.2)  **Dementia diagnosis:** NR  **Dementia type:** NR  **Dementia stage:** NR  **Acute illness:** Yes | **Design: CCT**  **Intervention: Type:** Dining environment & menu changes  **Provided by:** care home staff  **Details:** Int: improved dining atmosphere, readily available snacks, drinks machines, increased food choice, extended restaurant hours and self-service snacks all the time. Improved comfort during meals, increased choice at meal times, making eating with others a pleasurable and more sociable experience and encourage fading appetites.  **Control:** standard care (limited menu choice, fixed meal, drinks and snack times, no visitors joining residents for meal and crowded dining rooms)  **Duration of int/ follow up:** 1 year  **Compliance:** N/A  **Individualised:** No  **Ethics obtained:** Yes | **BMI, mean (SD)**  Int: 1st year 25.8(5.3), 2nd year 25.6 (4.8)  Cont: 1st year 24.7 (4.5), 2nd year 24.3 (4.8)  **Weight (kg, mean, SD)**  Int: 1st year 64.9(14.7), 2nd year 64.9 (15.4)  Cont: 1st year 62.3 (12.8), 2nd year 61.5 (14.6)  **Enjoyment of food & drink (mean change, SD)**  Int +0.28 (0.43),  Cont +0.09 (0.63)  P=0.24  **Residents appearing dehydrated (%, SD)**  Int: 1st year 7 (23.2), 2nd year 3 (10)  Cont: 1st year 10 (38.5), 2nd year 9 (34.6) | **MMSE (mean, SD)**  Int: 1st year 19(5.6), 2nd year 17 (6.2)  Cont: 1st year 17 (6.2), 2nd year 15 (7.9) | **Mortality: n (%)**  Int: 11 (19%)  Cont: 10 (20%) |
| **Robbins 2008 (32)**  **USA**  **Setting:** Hospitals & nursing homes  **Aim:**  To compare the effectiveness of two consistencies of thickened liquids on 3 month incidence of pneumonia in people with dementia or Parkinson disease | **Participants:** Patients from 47 acute care hospitals and 79 sub-acute residential facilities who aspirate.  **N: 515** (Int 1 Chin-tuck 259,  Int 2 thickened liquid, nectar-thick 133,  Int 3 thickened liquid, honey-thick 123)  **M/F:** 359/156  **Mean age:** 80.5 (SD NR)  **Nutritional status:** NR  **Cognitive status:** NR **Dementia diagnosis:** 70% had physician diagnosis of dementia. 260/ 515 (50%) diagnosed with dementia only while 255 had Parkinson’s disease with (n=101) or without dementia (n=154).  **Dementia type:** 88 AD, 75 vascular, 97 others  **Dementia stage:** NR  **Acute illness:** Yes | **Design:** RCT (parallel)  **Int: Type:** Dealing with aspiration (Modified diet & postural Ints)  **Provided by:** self or caregiver (NR)  **Details:** consuming thickened liquids (nectar thick or honey thick) in a head-neutral position. Liquids were manufactured for the study. Standardized recipes matching the viscosities of the barium products were developed for a wide variety of thickened beverages.  **Cont:** drinking all liquids in a chin-down posture  **Duration of Int/ follow up:** 3 mo  **Compliance: (for dementia patients)** Chin down 57% had >50% compliance, nectar-thick 73% had >50% compliance, hone-thick 91% had >50% compliance.  **Individualised:** No  **Ethics obtained:** Yes |  |  | **Aspiration pneumonia, 3 mo cumulative incidence:**  Int 1: 0.098  Int 2&3 combined: 0.116  HR= 0.84 (95% CI, 0.49 - 1.45); p=0.53  Int 2: 0.084  Int 3: 0.150  HR= 0.50 (0.23 - 1.09), p=0.083  **Pneumonia events**: 52, 32 in the 260 dementia patients. (Forest plot shows no statistically significant difference between Int 1 and Ints 2 & 3 for dementia patients). |
| **Salas-Salvado 2005 (35)**  **Spain**  **Setting:** 6 geriatric institutions  **Aim:** to evaluate the effect of whole formula diet on nutritional and cognitive status in AD patients | **Participants:** residents of 6 geriatric institutions, Spain, with severe cognitive impairment, advanced dementia or Alzheimer’s  **N:** 53 (Int 24) recruited; 38 (Int 15) completed study  **M/F: 5/19 (Int); 4/29 (Cont)**  **Mean age (SD):** Int.85.6 (6.6); Cont 83.9 (6.9).  **Nutritional status: BMI, kg/m2, mean (SD)**  Int. 22.3 (10.4),  Cont 21.7 (3.6)  **Cognitive status:** score ≥3 on Pfeiffer’s Cognitive Questionnaire **Dementia diagnosis:** DSM iv criteria  **Dementia type:** cognitive impairment, dementia or Alzheimer’s Disease  **Dementia stage:** severe; advanced AD  **Acute illness:** No | **Design:** RCT (parallel)  **Int: Type:** modification of food or drink; 3 packets/day Vegenat meal (450Kcals/pkt)  **Provided by:** caregivers  **Details:** complete diet based on natural lyophilised foods with liquid or semi-solid consistency; Dietetic advice for residents & staff  **Cont:**  Dietetic advice for residents & staff (though residents had severe cognitive impairment)  **Duration of Int/ follow up:** 3 mo  **Compliance:** NR  **Individualised:** No  **Ethics obtained:** yes | **Change in mean serum albumin, mg/dl, 0-3 months.**  Int (n=15): 3.76 (SD: 5.03);  Cont (n=23): 1.13 (SD: 5.7). p<0.05  **Change in mean weight, 0-3 mths.**  Int (n=15): 2.06 (SD: 1.9);  Cont (n=23): 0.32 (SD: 3.04). p<0.05  **Change in MNA, 0-3 months.**  Int (n=15): 3.6 (SD: 4.16);  Cont (n=23): 0.7 (SD: 5.05). Not significant | **Change in mean energy intake, 0-3 months.**  **Int (n=15)**  **+124 kcal/day (SD: 833);**  **Cont (n=23): -46 kcal/day (SD: 402)**  **Revman, p=0.46** | **Mortality:**  Int n=5  Cont n=3  **Pfeiffers test score, 0-3 months.**  Baseline: Int (n=15): 7.5 (SD; 2.5);  Cont (n=24):  7.8 (SD: 1.7).  3mths: Int (n=15): 7.0 (SD: 2.4);  Cont (n=23): 7.8 (SD: 1.8).  Reviewers analysis: p=0.27  **GDS_1_, no. scoring ≥5:**  Baseline, int n=15  Baseline, cont n=24  3 mth, int 15  3 mth, cont 23 |
| **Soltesz & Dayton 1995 (34)**  **US**  **Setting:** Alzheimer’s Care Centre  **Aim:** To determine the effect of menu changes (to include more finger foods) on AD residents’ weight and meal consumption patterns. | **Participants:** Care home residents with AD able to eat solid foods.  **N:** 43  **M/F:** 12/31  **Mean age (range):** 74 (60-97)  **Nutritional status, Weight, lbs, mean (SD):**  **Males:** 151.87 (18.88)  **Female:** 126.69 (21.83)  **Amount of food eaten, % of food served (both sexes):** 82.37 (SD NR)  **Cognitive status** NR **Dementia diagnosis:** residents of AD care facility  **Dementia type:** AD  **Dementia stage:** NR  **Acute illness:** NR | **Design:** BA  **Int: Type:** modification of food or drink  **Provided by:** care staff  **Details:** menu changes at care facility to include provision of finger foods  **Cont: N/A**  **Duration of Int/ follow up:** 6 mo  **Compliance:** NR  **Individualised:** no  **Ethics obtained:** NR | **Post Int. Body weight, lbs, mean (SD):**  Males: 151.93 (19.76)  Female: 128.41 (21.56) | **Pre Int,**  **Amount of food eaten, % of food served:** 82.37 (SD NR)  Post Int, amount of food eaten, % of food served  85.73 (SD NR)  Significant increase (p<0.05) reported |  |
| **Young 2005 (26,27)** | **As above** |  |  |  |  |
| **Eating or drinking assistance intervention studies** | | | | | |
| **Altus 2002 (41)**  **USA**  **Setting:**  Locked dementia unit  **Aim:**  To examine if changing the mode of meal delivery to “family-style” would increase residents’ communication and participation in mealtime tasks. | **Participants**: Dementia unit residents  **N:** 5  **M/F:** 0/5  **Mean age (range)**: 80 (76-87)  **Nutritional status:** NR  **Cognitive status: mean** MMSE score (range) 8 (3-16)  **Dementia diagnosis:** physician diagnosis  **Dementia type:** AD or other dementia  **Dementia stage:** Moderate to severe  **Acute illness:** NR | **Design: BA** (time-series, repeated measures)  **Int**: Family- style meals (with or without nursing assistant training)  **Details: ABAB’ design**  **Provided by:** health worker/care home staff  Details: **Cont 1** (baseline 10 d) **Int 1** (family style meals 5 d) **Cont 2** (repeat 5d) **Int 2** (family style + assistant training 5d)  **Cont**: pre-plated meals  **Duration of Int/ follow up**: 25 days (10 int days/25 days)  **Compliance:** NR  **Individualised:** No  **Ethics obtained:** NR |  | **CNA’s satisfaction with level of participation:**  post int: 5 (very satisfied) vs pre int: 2 (somewhat satisfied) | **Participation in mealtime tasks: (% participation)**  Int 1 24%, Cont 1 10%  Int 2 65%, Cont 2 6% 2  **Appropriate communication:** (% intervals with appropriate communication)  Int 1 10.6%, Cont 1 5.5%  Int 2 17.9%, Cont 2 3.8%  **Praise statements** by CNA  Int 1 7.2/meal, Cont 1 0.2/meal  Int 2 14.2/meal, Cont 2 0/meal |
| **Charras & Fremontier 2010 (42)**  **France**  **Setting**  **2 Dementia units in nursing homes**  **Aim:**  To study the impact of changed mealtime experience for people with AD | **Participants**: AD patients from 2 special units  **N:** 18 (8 Int, 10 Cont)  **M/F:** Not reported  **Mean age (SD)**:85.2 (6.5)  **Nutritional status:** only weight reported  **Cognitive status: mean MMSE score (SD)** 7.5 (5.6) **Dementia diagnosis:** diagnosed (no details)  **Dementia type:** AD  **Dementia stage:** Severe  **Acute illness:** NR | **Design: CCT (cluster)**  **Int**: Type: Dining environment (shared mealtime meals)  **Provided by:** Nursing home staff  Details: Shared lunchtime meals between staff & residents with individual help available when necessary. 2 large tables of 8 or 9 patients and staff. 4 courses offered.  **Cont**: Usual care  12 session training programme + environmental design to increase orientation & wellbeing were implemented in both Int and Cont units.  **Duration of Int/ follow up**: 3 mo  **Compliance:** NR  **Individualised:** No  **Ethics obtained:** NR | **Body weight, kg, mean (SD)**  Int: Baseline 51.3 (8.7), post Int 54.6 (9)  Cont: baseline 63 (12.6), post Int 60.8 (12.4)  int +3.4 kg vs Cont -2.2 kg, p<0.0244 |  | **Autonomy (qualitative observations):**  Some trying to eat independently  Residents serving themselves**,** helping in clearing up  **Interaction quality**  Increase resident-resident & staff-resident interaction.  Increased staff awareness of residents’ likes, dislikes, biography.  Residents helpful to each other and increased talking.  **Attitudes:** Less resident wandering  More satisfaction and effort by staff.  **Food quality:**  Increased as staff reported back  **Timing:**  Meals took 1-1.5 hrs, presenting a challenge |
| **Huang 2009 (43)**  **Taiwan**  **Setting**  **Older person care facility**  **Aim:**  To help older people with dementia in a home by applying reminiscence group work. | **Participants**: Nursing home dementia residents  **N:** 12  **M/F:** 7/4  **Age**: 4<74 yr, 3=75-84, 4>85yr  **Nutritional status:** NR  **Cognitive status: MMSE, mean (SD)**  15.9 (5) **Dementia diagnosis:** diagnosed (no details)  **Dementia type:** NR  **Dementia stage:** Mild to moderate  **Acute illness:** NR | **Design: BA**  **Int**: Type: Reminiscence therapy  **Provided by:** Researchers?  **Details**: 8 sessions of reminiscence cooking lessons of dishes remembered by participants. Each session; 10 min warm up, 20 min cooking, 30 min eating and sharing results. After each session participants interviewed (30 min)  **Cont**: N/A  **Duration of Int/ follow up**: 8 weeks  **Compliance:** NR  **Individualised:** yes  **Ethics obtained:** NR |  |  | **PIS- happiness subscale (mean, SD)**  Pre 4.6(0.92), post 5.8(0.4) p=0.01  **PIS- positive communication subscale (mean, SD)**  Pre 4.7(1.19), post 5.6 (0.66) p=0.05  **PFS:** all items improved except feeling emotions  **MMSE (mean, SD)**  Pre 15.9 (5),  post 17.1(4) (p>0.05) |
| **Pietro & Boczko 1998 (44)**  **USA**  **Dementia unit within a nursing home**  **Aim:**  To document the effectiveness of the breakfast club, a multi modality group communication therapy for AD patients to compare its outcomes with those of standard conventional group therapy for institutionalised patients with mid-stage AD. | **Participants:** Institutionalised patients with mid-stage dementia  **N:** (Int 20, Cont 20)  **M/F:** NR  **Mean age (SD):**  Int. 84.6 (4.7), Cont 86.2 (6.03)  **Cognitive status, MMSE, mean (SD)** Int. 15.6 (4.0), Cont (n=18) 13.8 (4.8)  **Dementia diagnosis:** NR  **Dementia type:** AD  **Dementia stage:** mild and moderate AD  **Acute illness:** NR | **Design: CCT**  **Int: Type:** Facilitated breakfast club (environmental improvements, social activities including food and drink, prompting)  **Provided by:** health worker (speech & language therapist)  **Details:** groups of 5 residents sat around a table and prepared, ate and cleared up breakfast, facilitated by researcher, who encouraged conversation about the tasks.  **Cont:** groups of 5 residents sat around a table, coffee was served, conversation facilitated by researcher  **Duration of Int/ follow up:** 45 mins each morning/5 days per wk for 12 wks  **Compliance:** NR  **Individualised:** No  **Ethics obtained:** NR |  |  | **Interest & involvement:**  Int: 12.0  Cont: NR  p<0.0005  **Procedural memory score:**  Int: 6.8  Cont: NR  p<0.0005  **COMFI, mean change:**  Int: 16.05 (SD: 8.22)  Cont: 0.5 (SD: 4.77)  p=<0.00001 (calculated by reviewers)  **ABCD.**  Int: 10.87 (SD: N/R)  Cont: 10.25 (SD: N/R)  **Views of participants, carers and family**  Int: participants remembered each other’s names; increased response to non-verbal cues, increased use of humour, use of empathetic statements, spontaneous singing; decreased distractibility and wandering |
| **Simmons 2001 (40)**  **USA**  **Setting:** 2 Nursing Homes  **Aim:** To evaluate a three-phase behavioural intervention to improve fluid intake in nursing home residents**.** | **Participants:** incontinent residents from two community nursing homes  **N:** Int 48, Cont 15  **M/F:** Int: 4/44; Cont: 5/10  **Mean age (SD):** Int. 88.7 (7.1); Cont 86.3 (6.1)  **Nutritional status, total mealtime food & fluid intake, % (SD) of portion served:**  Int 53.8 (15.6); Cont 58.9 (14.7)  **Hydration status (n=32), serum osmolality, mOsmol/kg, mean (SD):** Int 303.6 (9.1); Cont 303.4 (8.5)  **BUN: creatinine ratio, mean (SD):** Int 21.7 (6.1); Cont 24.0 (4.6)  **Cognitive status, MMSE, mean (SD):** Int 12.1 (7.9); Cont 13.9 (6.5) **Dementia diagnosis:** Diagnosis of dementia or MMSE score (assessed by researchers)  **Dementia type:** NR  **Dementia stage:** NR  **Acute illness:** NR | **Design: CCT**  **Int:** Behavioural  **Provided by:** Research Staff  **Details:** Weeks 1-16 (phase 1): residents prompted to exercise every two hours (7.00am–3.30pm) and checked for incontinence, offered toileting assistance and drinks.  Weeks 17-24 (phase 2): increased to eight prompts/day  Weeks 25-32 (phase 1): this was supplemented by an increased choice of drinks  **Cont:** Usual care (not described)  **Duration of Int/ follow up:** 32 weeks  **Compliance:** Phase 1: mean number of prompts (both groups combined) = 3.8/day (SD: 0.2); Phases 2&3: 7.6/day (SD: 0.4)  **Individualised:** No  **Ethics obtained:** Yes | **Serum osmolality, mOsmol/kg, mean (SD):**  Int: 297.0 (10.8);  Cont: 294.7 (11.9). p=0.57  **BUN:creatinine ratio, mean (SD):**  Int: 22.9 (5.6);  Cont: 23.8 (7.2), p=0.71  p values calculated by reviewers | **Total mealtime food & fluid intake, % (SD) of portion served:**  Phase 1, assessed at 8 wks:  Int: 52.0 (17.5);  Cont: 58.8 (15.6)  **Phase 3, assessed at 32 weeks:**  Int: 53.1 (19.6);  Cont: 57.3 (17.7)  p=0.43 (calculated by reviewers)  **Between-meal fluid intake, floz/day:**  Phase 1:  Int: 9.8 (4.6);  Cont: N/R  Phase 2:  Int: 16.1 (10.0);  Cont: N/R  Phase 3:  Int: 21.4 (12.7);  Cont: N/R |  |
| **Simmons 2008 (38)**  **USA**  **Skilled nursing homes**  **Aim: To determine the effects of a feeding assistance Int on food and fluid intake and body weight.**  *(nb: This paper reports on a 48-week crossover trial, but at the crossover point, residents who were excluded from the trial initially were re-assessed for inclusion. Thus, this review is just considering those participating in the initial 24-week trial, described by reviewers as a parallel RCT, n=24)* | **Participants**: residents of 4 NH with low food and fluid intake and responsive to interventions.  **N:** 69  **M/F:** Int: 83.6% female; Cont: 85.7% female  **Mean age, sd**: Int: 82.3 (SD: 11.9); Cont: 83.5 (11.5)  **Nutritional status, BMI, Kg/m^2^, n (%) with BMI <20.**  Int 12 (19.7), Cont 9 (14.3)  **E intake, kcal/day, mean (SD):**  Int 1237 (302), Cont 1189 (398)  **Cognitive status, MMSE, mean (SD) score.**  Int: 15.4 (8.8); Cont: 15 (8.6) **Dementia diagnosis:**  MMSE score administered by researchers  **Dementia type:** N/R  **Dementia stage:** N/R  **Acute illness:** NR  NB – baseline characteristics provided for the larger study group only (n=124), not for the sub-study (n=69) described here. | **Design: RCT (cluster parallel)**  **Int**: Type: feeding assistance and food service  **Provided by:** researchers  **Details**:  Participants randomised to Cont group or one of two Ints (meal-time or between-meal Int, dependant on their response to a pre-study 2-day trial for each type).  1. Individual meal-time assistance (1 staff member per resident) for 2 meals/day. Assistance included proper positioning, compliance with dining room preferences, optional tray substitutions, appropriate prompting.  2. Between-meal carts offering food and drinks x2/day + appropriate assistance.  **Cont**:  Usual care (not described)  **Duration of Int/ follow up**: 5 days/week, 24 weeks*.*  **Compliance: measured but not reported**  **Individualised: yes**  **Ethics obtained: yes** | **Results not reported for n=69.**  **Results for those taking part in the 48-week crossover trial, n=124**  Final BMI in Int group, compared to Cont = +0.72 units, *p=*0.09  Final weight in Int group compared to Cont group = +4.0 lbs | **Results reported for n=64 (those completing the initial 24-week trial period)**  **Energy intake, kcal/day, mean (SD):**  Int: 1539 (322)  Cont: 1316 (476) | **Mortality: N/R**  **Resource use; NH staff time**  **Int N/R**  **Cont** <10 mins/meal/resident**.**  **Resource use (for 48-wk crossover)**  **Int**  1.Staff assistance/ resident/meal, mins, mean (SD):  42.2 (19.1).  87% of care episodes were provided 1:1  2. Staff assistance/ resident/snack, mins, mean (SD): 13.9 (10.3).  76% of care episodes were provided 1:1  **Cont**  Staff assistance averaged <10 minutes/meal/ resident |
| **Simmons 2010 (22)** | **As above** |  |  |  |  |
| **Wong 2008 (39)**  **New Zealand**  **Setting**  Short stay assessment unit  **Aim:**  To evaluate strategies designed to improve nutrition in elderly hospitalised patients with dementia. | **Participants**: Elderly dementia In-patients in a short stay unit  **N:** Phase 3: n = 7  **M/F:** P3: 5/2,  **Mean age (SD)**: P3: 77.0 (6.5)  **Nutritional status: BMI, mean (SD)** P3: 24.3 (3.5),  **Cognitive status:** NR **Dementia diagnosis:** dementia unit patients (no details)  **Dementia type:** NR  **Dementia stage:** NR  **Acute illness:** yes | **Design:** BA (Interrupted time series)  **Int**: Type: P3 (feeding assistance)  **Provided by: NR**  Details: Each phase lasted 12 weeks followed by a 5 week gap to ensure new patients in each phase.  **P3:** Maximising food and fluid intake at mealtimes by using volunteers to feed patients.  **Cont**: P1 observation only  **Duration of Int/ follow up**: 12 weeks  **Compliance:** NR  **Individualised:** No  **Ethics obtained:** Yes | **BMI, Kg/m2, mean change:**  P1: -0.6 (p<0.001).  P3: +0.37 (p<0.04)  **Mid arm circumference (MAC), cm, mean (SD)**  P1: not measured  P3: +0.14 (0.24)  (p= NS) | **Energy intake, Kcal, mean change**  **P3: +44.1 (p<0.001).** |  |

AD= Alzheimer’s dementia,

ADAS-cog= Alzheimer’s Disease Assessment Scale - cognitive subscale (which scores from 0=no impairment to 75= severe impairment),

ADCS-ADL= and ADL scoring system

ADL= Activities of daily living,

BA = before/after

BI=Barthel Index (functional capacity scale of 0-20, higher score=better functional capacity),

BMI = body mass index, weight in kg divided by height in m squared, kg/m^2^

CCT= controlled clinical trial,

CDR= Clinical Dementia Rating scale (0=normal, 0.5-4 questionable cognitive impairment, 4.5-9 mild dementia, 9.5-15.5 moderate, ≥16 Severe dementia)

CHO= Carbohydrates,

CPS = Cognitive performance scale (scale of 0-6 with 0 intact & 6 very severe impairment)
E = energy

EBS= Eating Behaviour Scale,

EdFed= Edinburgh feeding,

F=female,

GDS= Global Deterioration Scale,

GDS_1_ = geriatric depression scale (score >5points suggests depression and warrants comprehensive assessment, ≥10 is almost always Int= intervention, indicative of depression),

ITT = intention to treat

M= male,

MAC = mid arm circumference

MAMC = mid-upper arm muscle circumference

MCI = mild cognitive impairment

MMSE= Mini mental State Examination,

MNA= Mini Nutritional Assessment,

MOCA = Montreal Cognitive Assessment (scale 0-30),

MUAC = mid upper arm circumference

NH= Nursing home,

NR= Not reported,

ONS= oral nutritional supplement,

PFS = Participatory Feeling Scale (4 items, including feelings of emotion, stress relief, adaptation and impression),

PIS = Personal Interaction Scale

SD = standard deviation,

SDAT= Senile disease of Alzheimer type,

RCT= Randomised controlled trial,

TSF = triceps skinfold thickness

UAC = upper arm circumference

WMS-r delayed or immediate verbal recall = a form of cognitive assessment

**References**

1. Abalan F, Manciet G, Dartigues JF, Decamps A, Zapata E, Saumtally B, Galley P. Nutrition and SDAT. Biol Psychiatry 1992;31(1):103-5.

2. Beck AM, Ovesen L, Schroll M. Home-made oral supplement as nutritional support of old nursing home residents, who are undernourished or at risk of undernutrition based on the MNA. A pilot trial. Mini Nutritional Assessment. Aging clinical and experimental research 2002;14(3):212-5.

3. Carlsson M, Gustafson Y, Haglin L, Eriksson S. The feasibility of serving liquid yoghurt supplemented with probiotic bacteria, Lactobacillus rhamnosus LB 21, and Lactococcus lactis L1A - A pilot study among old people with dementia in a residential care facility. The journal of nutrition, health & aging 2009;13(9):813-9.

4. Carlsson M, Littbrand H, Gustafson Y, Lundin-Olsson L, Lindelof N, Rosendahl E, Haglin L. Effects of high-intensity exercise and protein supplement on muscle mass in adl dependent older people with and without malnutrition-a randomized controlled trial. The journal of nutrition, health & aging 2011;15(7):554-60.

5. Rosendahl E, Lindelöf N, Littbrand H, Yifter-Lindgren E, Lundin-Olsson L, Håglin L, Gustafson Y, Nyberg L. High-intensity functional exercise program and protein-enriched energy supplement for older persons dependent in activities of daily living: a randomised controlled trial. The Australian journal of physiotherapy [Randomized Controlled Trial; Research Support, Non-U.S. Gov't] 2006;52:105-13. Internet: <http://onlinelibrary.wiley.com/o/cochrane/clcentral/articles/780/CN-00565780/frame.html>

6. Carver AD, Dobson AM. Effects of dietary supplementation of elderly demented hospital residents. J Hum Nutr Diet 1995;8(6):389-94.

7. de Sousa OLV, Amaral TF. Three-week nutritional supplementation effect on long-term nutritional status of patients with mild Alzheimer disease. Alzheimer Dis Assoc Disord 2012;26(2):119-23. doi: 10.1097/WAD.0b013e31822c5bb3.

8. Faxen-Irving G, Andren-Olsson B, af Geijerstam A, Basun H, Cederholm T. The effect of nutritional intervention in elderly subjects residing in group-living for the demented. Eur J Clin Nutr 2002;56(3):221-7.

9. Fiatarone Singh MA, Bernstein MA, Ryan AD, O'Neill EF, Clements KM, Evans WJ. The effect of oral nutritional supplements on habitual dietary quality and quantity in frail elders. The journal of nutrition, health & aging 2000;4(1):5-12.

10. Gregorio PG, Diaz SPR, Casado JMR. Dementia and Nutrition. Intervention study in institutionalized patients with Alzheimer Disease. The journal of nutrition, health & aging 2003;7(5):304-8.

11. Krikorian R, Nash TA, Shidler MD, Shukitt-Hale B, Joseph JA. Concord grape juice supplementation improves memory function in older adults with mild cognitive impairment. Br J Nutr [Randomized Controlled Trial; Research Support, Non-U.S. Gov't] 2010;103:730-4. Internet: <http://onlinelibrary.wiley.com/o/cochrane/clcentral/articles/937/CN-00728937/frame.html>

12. Krikorian R, Shidler MD, Nash TA, Kalt W, Vinqvist-Tymchuk MR, Shukitt-Hale B, Joseph JA. Blueberry supplementation improves memory in older adults. J Agric Food Chem 2010;58(7):3996-4000.

13. Krikorian R, Boespflug EL, Fleck DE, Stein AL, Wightman JD, Shidler MD, Sadat-Hossieny S. Concord grape juice supplementation and neurocognitive function in human aging. J Agric Food Chem 2012;60(23):5736-42.

14. Lauque S, Arnaud-Battandier F, Mansourian R, Guigoz Y, Paintin M, Nourhashemi F, Vellas B. Protein-energy oral supplementation in malnourished nursing-home residents. A controlled trial. Age Ageing [Clinical Trial; Randomized Controlled Trial] 2000;29:51-6. Internet: <http://onlinelibrary.wiley.com/o/cochrane/clcentral/articles/408/CN-00275408/frame.html>

15. Lauque S, Arnaud-Battandier F, Gillette S, Plaze JM, Andrieu S, Cantet C, Vellas B. Improvement of weight and fat-free mass with oral nutritional supplementation in patients with Alzheimer's disease at risk of malnutrition: a prospective randomized study. Journal of the American Geriatrics Society [Clinical Trial; Randomized Controlled Trial; Research Support, Non-U.S. Gov't] 2004;52:1702-7. Internet: <http://onlinelibrary.wiley.com/o/cochrane/clcentral/articles/736/CN-00491736/frame.html>

16. Manders M, De Groot LC, Hoefnagels WH, Dhonukshe-Rutten RA, Wouters-Wesseling W, Mulders AJ, Van Staveren WA. The effect of a nutrient dense drink on mental and physical function in institutionalized elderly people. The journal of nutrition, health & aging 2009;13(9):760-7.

17. Navratilova M, Jarkovsky J, Ceskova E, Leonard B, Sobotka L. Alzheimer disease: Malnutrition and nutritional support. Clin Exp Pharmacol Physiol 2007;34(SUPPL. 1):S11-S3.

18. Pivi GA, da Silva RV, Juliano Y, Novo NF, Okamoto IH, Brant CQ, Bertolucci PH. A prospective study of nutrition education and oral nutritional supplementation in patients with Alzheimer's disease. Nutr J 2011;10:98.

19. Planas M, Conde M, Audivert S, Perez-Portabella C, Burgos R, Chacon P, Rossello J, Boada M, Tarraga LL. Micronutrient supplementation in mild Alzheimer disease patients. Clinical nutrition (Edinburgh, Scotland) [Research Support, Non-U.S. Gov't] 2004;23:265-72. Internet: <http://onlinelibrary.wiley.com/o/cochrane/clcentral/articles/008/CN-00487008/frame.html>

20. Scheltens P, Kamphuis PJ, Verhey FR, Olde Rikkert MG, Wurtman RJ, Wilkinson D, Twisk JW, Kurz A. Efficacy of a medical food in mild Alzheimer's disease: A randomized, controlled trial. Alzheimers Dement [Clinical Trial; Multicenter Study; Randomized Controlled Trial; Research Support, Non-U.S. Gov't] 2010;6:1-10.e1. Internet: <http://onlinelibrary.wiley.com/o/cochrane/clcentral/articles/006/CN-00742006/frame.html>

21. Scheltens P, Twisk JW, Blesa R, Scarpini E, Arnim CA, Bongers A, Harrison J, Swinkels SH, Stam CJ, Waal H, et al. Efficacy of Souvenaid in mild Alzheimer's disease: results from a randomized, controlled trial. Journal of Alzheimer's disease : JAD [Multicenter Study; Randomized Controlled Trial; Research Support, Non-U.S. Gov't] 2012;31:225-36. Internet: <http://onlinelibrary.wiley.com/o/cochrane/clcentral/articles/961/CN-00871961/frame.html>

22. Simmons S, Zhuo X, Keeler E. Cost-effectiveness of nutrition interventions in nursing home residents: A pilot intervention. J Nutr Health Aging 2010;14(5):367-72. doi: 10.1007/s12603-010-0082-1.

23. Stange I, Bartram M, Liao Y, Poeschl K, Kolpatzik S, Uter W, Sieber CC, Stehle P, Volkert D. Effects of a Low-Volume, Nutrient- and Energy-Dense Oral Nutritional Supplement on Nutritional and Functional Status: A Randomized, Controlled Trial in Nursing Home Residents. Journal of the American Medical Directors Association 2013;14(8):628.e1-8. doi: 10.1016/j.jamda.2013.05.011.

24. Wouters-Wesseling W, Wouters AE, Kleijer CN, Bindels JG, de Groot CP, van Staveren WA. Study of the effect of a liquid nutrition supplement on the nutritional status of psycho-geriatric nursing home patients. Eur J Clin Nutr 2002;56(3):245-51.

25. Wouters-Wesseling W, Slump E, Kleijer CN, de Groot LC, van Staveren WA. Early nutritional supplementation immediately after diagnosis of infectious disease improves body weight in psychogeriatric nursing home residents. Aging clinical and experimental research 2006;18(1):70-4.

26. Young KW, Greenwood CE, Reekum R, Binns MA. Providing nutrition supplements to institutionalized seniors with probable Alzheimer's disease is least beneficial to those with low body weight status. Journal of the American Geriatrics Society [Clinical Trial; Randomized Controlled Trial; Research Support, Non-U.S. Gov't] 2004;52:1305-12. Internet: <http://onlinelibrary.wiley.com/o/cochrane/clcentral/articles/003/CN-00481003/frame.html>

27. Young KW, Greenwood CE, Reekum R, Binns MA. A randomized, crossover trial of high-carbohydrate foods in nursing home residents with Alzheimer's disease: associations among intervention response, body mass index, and behavioral and cognitive function. J Gerontol A Biol Sci Med Sci [Clinical Trial; Randomized Controlled Trial; Research Support, Non-U.S. Gov't] 2005;60:1039-45. Internet: <http://onlinelibrary.wiley.com/o/cochrane/clcentral/articles/764/CN-00529764/frame.html>

28. Beck AM, Damkjaer K, Sørbye LW. Physical and social functional abilities seem to be maintained by a multifaceted randomized controlled nutritional intervention among old (>65 years) Danish nursing home residents. Archives of gerontology and geriatrics [Randomized Controlled Trial; Research Support, Non-U.S. Gov't] 2010;50:351-5. Internet: <http://onlinelibrary.wiley.com/o/cochrane/clcentral/articles/403/CN-00752403/frame.html>

29. Boffelli S, Rozzini R, Trabucchi M. Nutritional Intervention in Special Care Units for Dementia. Journal of the American Geriatrics Society 2004;52(7):1216-7. doi: 10.1111/j.1532-5415.2004.52327_1.x.

30. Bautmans I, Demarteau J, Cruts B, Lemper JC, Mets T. Dysphagia in elderly nursing home residents with severe cognitive impairment can be attenuated by cervical spine mobilization. J Rehabil Med [Comparative Study; Randomized Controlled Trial] 2008;40:755-60. Internet: <http://onlinelibrary.wiley.com/o/cochrane/clcentral/articles/364/CN-00651364/frame.html>

31. Germain I, Dufresne T, Gray-Donald K. A novel dysphagia diet improves the nutrient intake of institutionalized elders. Journal of the American Dietetic Association 2006;106(10):1614-23. doi: 10.1016/j.jada.2006.07.008.

32. Robbins J, Gensler G, Hind J, Logemann JA, Lindblad AS, Brandt D, Baum H, Lilienfeld D, Kosek S, Lundy D, et al. Comparison of 2 interventions for liquid aspiration on pneumonia incidence: A randomized trial. Ann Intern Med 2008;148(7):509-18.

33. Jean LA. "Finger food menu" restores independence in dining. Health care food & nutrition focus 1997;14(1):4-6.

34. Soltesz KS, Dayton JH. The effects of menu modification to increase dietary intake and maintain the weight of Alzheimer residents. American journal of Alzheimer's disease and other dementias 1995;10(6):20-3. doi: 10.1177/153331759501000604.

35. Salas-Salvado J, Torres M, Planas M, Altimir S, Pagan C, Gonzalez ME, Johnston S, Puiggros C, Bonada A, Garcia-Lorda P. Effect of oral administration of a whole formula diet on nutritional and cognitive status in patients with Alzheimer's disease. Clinical nutrition (Edinburgh, Scotland) 2005;24(3):390-7.

36. Keller HH, Gibbs AJ, Boudreau LD, Goy RE, PattiIlo MS, Brown HM. Prevention of Weight Loss in Dementia with Comprehensive Nutritional Treatment. Journal of the American Geriatrics Society 2003;51(7):945-51. doi: 10.1046/j.1365-2389.2003.51307.x.

37. Kenkmann A, Price G, Bolton J, Hooper L. Health, wellbeing and nutritional status of older people living in UK care homes: an exploratory evaluation of changes in food and drink provision. BMC Geriatrics 2010;10(1):28.

38. Simmons SF, Keeler E, Zhuo X, Hickey KA, Sato HW, Schnelle JF. Prevention of unintentional weight loss in nursing home residents: A controlled trial of feeding assistance. Journal of the American Geriatrics Society 2008;56(8):1466-73.

39. Wong A, Burford S, Wyles CL, Mundy H, Sainsbury R. Evaluation of strategies to improve nutrition in people with dementia in an assessment unit. The journal of nutrition, health & aging 2008;12(5):309-12.

40. Simmons SF, Alessi C, Schnelle JF. An intervention to increase fluid intake in nursing home residents: prompting and preference compliance. Journal of the American Geriatrics Society [Clinical Trial; Multicenter Study; Randomized Controlled Trial; Research Support, U.S. Gov't, P.H.S.] 2001;49:926-33. Internet: <http://onlinelibrary.wiley.com/o/cochrane/clcentral/articles/247/CN-00350247/frame.html>

41. Altus DE, Engelman KK, Mathews RM. Using family-style meals to increase participation and communication in persons with dementia. Journal of gerontological nursing 2002;28(9):47-53.

42. Charras K, Fremontier M. Sharing meals with institutionalized people with dementia: a natural experiment. J Gerontol Soc Work 2010;53(5):436-48.

43. Huang SL, Li CM, Yang CY, Chen JJ. Application of reminiscence treatment on older people with dementia: a case study in Pingtung, Taiwan. J Nurs Res 2009;17(2):112-9.

44. Pietro MJS, Boczko F. The Breakfast Club: Results of a study examining the effectiveness of a multi-modality group communication treatment. American journal of Alzheimer's disease and other dementias 1998;13(3):146-58. doi: 10.1177/153331759801300307.
