# Supplementary material for: Effectiveness of interventions to directly support food and drink intake in people with dementia: systematic review and meta-analysis
Source: BMC Geriatr. 2016 Jan 22;16:26. doi: 10.1186/s12877-016-0196-3 (PMC4722767; doi:10.1186/s12877-016-0196-3)

**Supplementary File 3: Summary of study validity and further meta-analysis results of direct interventions**

**EDWINA (Eating and Drinking Well IN dementiA)**

**Supplementary Figure 3.1. Risk of bias summary for studies of oral supplementation**

**
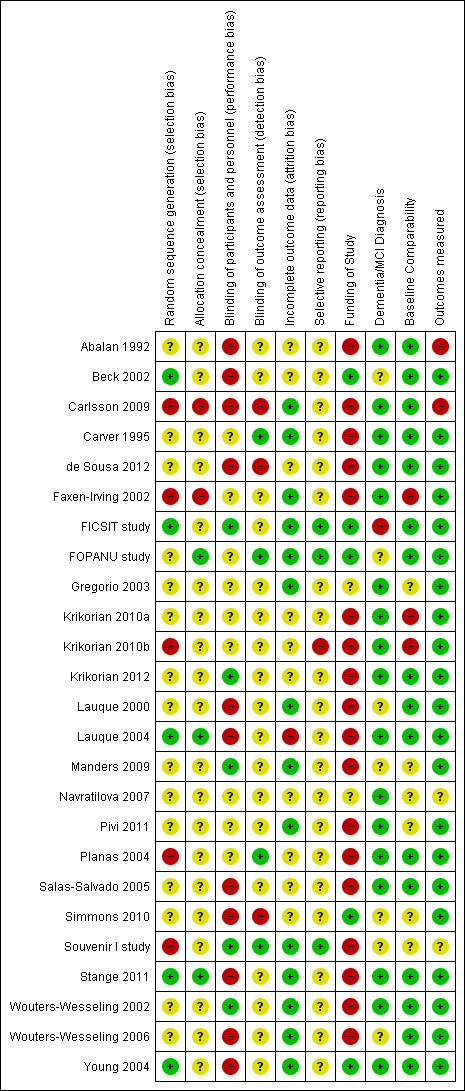
**

Note: A study was considered at low risk of bias overall where they were at low risk of both selection bias and detection bias (so had a green plus in the first, second and fourth columns above). Planas 2004 and Souvenir I were assessed as before-after studies, as they were used within this review – they were set up as RCTs. These are the criteria used to assess the elements of validity within included studies:

- Random sequence generation: Was there selection bias (biased allocation to interventions) due to *inadequate generation of a randomised sequence*?
- Allocation concealment: Was there selection bias (biased allocation to interventions) due to *inadequate concealment of allocations prior to assignment*?
- Blinding of participants and personnel: Was there performance bias due to knowledge of the allocated interventions by participants and personnel during the study?
- Blinding of outcome assessment: Was there detection bias due to knowledge of the allocated interventions by outcome assessors?
- Incomplete outcome data: Was there attrition bias due to amount, nature or handling of incomplete outcome data?
- Selective reporting: Was there reporting bias due to selective outcome reporting?
- Funding of study: Was there bias due to commercial funding/ involvement?
- Dementia or MCI diagnosis: Was there bias due to dementia/ mild cognitive impairment diagnosis not reported in accordance with recognised criteria?
- Baseline comparability: Was there bias due to significant baseline differences between study groups?
- Outcomes measured: Was there bias due to selected outcome measures not being suitable to reflect an improvement?

**Supplementary Figure 3.2. Forest plot, effects of oral nutrition supplement on the mini-nutrition assessment (MNA) in RCTs**


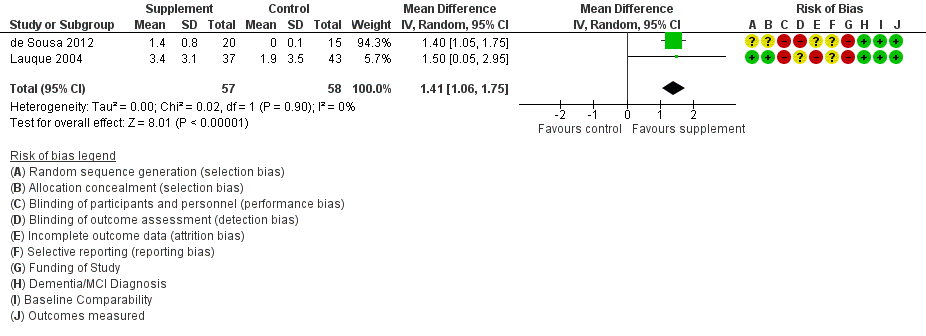


**Supplementary Figure 3.3. Forest plot, effects of oral nutrition supplement on energy intake (in kcal/day) in RCTs**


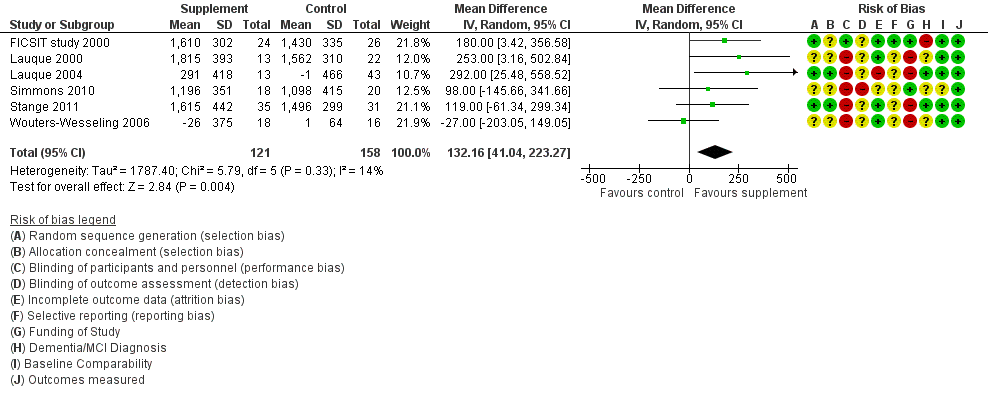


**Supplementary Figure 3.4. Forest plot, effects of oral nutrition supplement on mortality in RCTs**


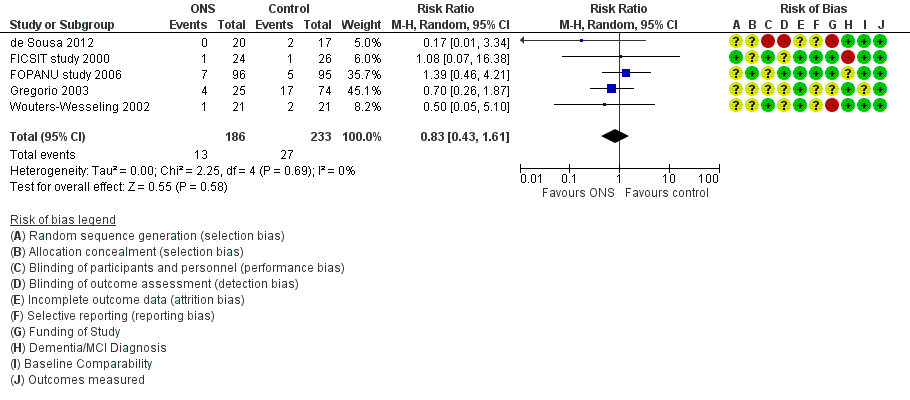


**Supplementary Figure 3.5. Risk of bias summary for interventions for swallowing problems**

**
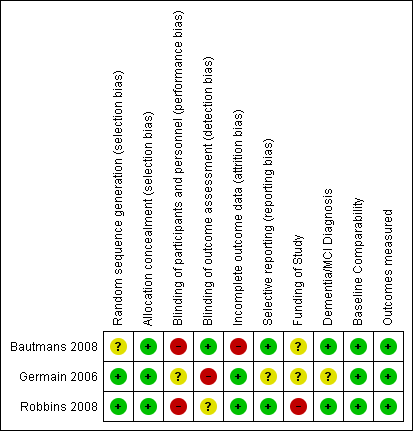
**

**Supplementary Figure 3.6. Risk of bias summary for interventions assessing effects of food modification**

**
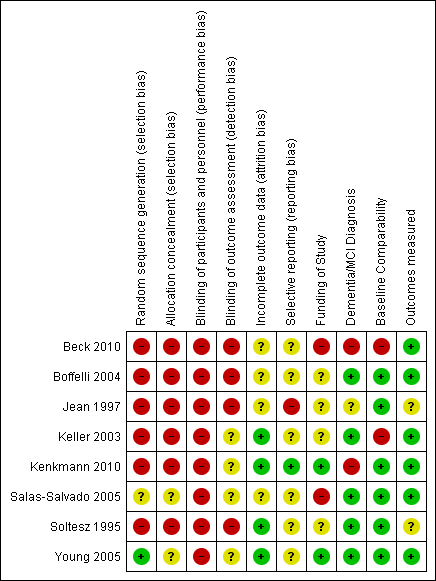
**

**Supplementary Figure 3.7. Risk of bias summary for interventions assessing effects of assistance with eating and drinking**


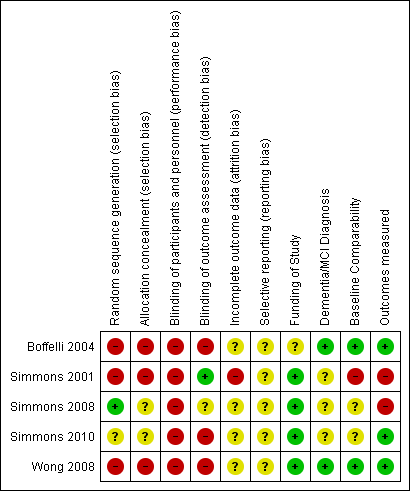


**Supplementary Figure 3.8. Risk of bias summary for interventions with a strong social element**


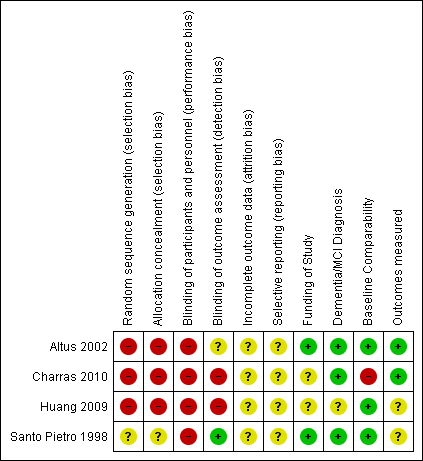

Supplement: Additional file 3: — Summary of study validity and further meta-analysis results of direct interventions. (DOCX 136 kb) [file 12877_2016_196_MOESM3_ESM.docx]
